# Mitochondrial quality control in neurodegenerative diseases: from molecular mechanisms to natural product therapies

**DOI:** 10.3389/fphys.2025.1695681

**Published:** 2025-10-16

**Authors:** Boxun Chen, Qing Wang, Yannan Wang, Qingzhi Liu, Weiyue Chen, Hong Mao, Jiamin Li, Qi Liu, Xue Zhou

**Affiliations:** ^1^ Department of First Clinical Medical College, Shandong University of Traditional Chinese Medicine, Jinan, China; ^2^ Beijing University of Chinese Medicine, Beijing, China; ^3^ Affiliated Hospital of Shandong University of Traditional Chinese Medicine, Jinan, China

**Keywords:** neurodegenerative diseases, mitochondrial quality control, molecular mechanisms, natural products, toxicology and adverse effects

## Abstract

**Background:**

Neurodegenerative diseases, such as Alzheimer’s disease, Parkinson’s disease, *etc.*, are a group of complex and heterogeneous disorders characterized by progressive synaptic loss and pathological protein alterations. Mitochondria are the main source of energy produced by neurons and support the high energy consumption of the nervous system. Mitochondrial quality control, involving processes like mitophagy and mitochondrial biogenesis, is crucial for mitochondrial homeostasis, and mitochondrial dysfunction is closely related to neurodegenerative diseases pathogenesis, making targeting mitochondrial quality control a potential therapeutic strategy. Natural products offer benefits such as cost-effectiveness, fewer side effects, and other positive qualities, making them suitable choices as supplements or alternatives to traditional drugs for treating neurodegenerative diseases.

**Methods:**

A thorough search was conducted on many databases including Web of Science, PubMed, EMBASE, and MEDLINE to investigate the role of mitochondria in neurodegenerative diseases and the therapeutic effects of natural products.

**Results:**

By searching the relevant studies on neurodegenerative diseases and mitochondria in recent years, we observed a rise in the number of studies examining the functional characteristics and biological events of mitochondrial quality control systems in neurodegenerative diseases pathogenesis and the potential for natural products regulating mitochondrial quality control to improve neurodegenerative diseases.

**Conclusion:**

This review summarizes the functional characteristics and biological events of mitochondrial quality control systems in neurodegenerative diseases pathogenesis, and comprehensively analyzes the pharmacological mechanisms by which natural products regulate mitochondrial quality control to improve neurodegenerative diseases, aiming to provide a scientific basis for further research and new clinical drug development.

## 1 Introduction

Neurodegenerative diseases (NDs) represent a complex and heterogeneous group of disorders, including Alzheimer’s disease (AD), Parkinson’s disease (PD), Huntington’s disease (HD), and amyotrophic lateral sclerosis (ALS). These conditions are characterized by the selective loss and progressive degeneration of neurons and glial cells within the nervous system. Clinically, they manifest as impairments in cognitive and behavioral functions, which may ultimately lead to mortality (Li et al., 2023). NDs represent a significant global health concern. Due to the aging population worldwide, the incidence and mortality rates associated with NDs are on the rise (Ferri et al., 2005). Due to the pathophysiological mechanisms of NDs involving the cross-regulation of multiple molecular pathways, and given that the exact etiology of most NDs remains unclear, these conditions are primarily associated with mechanisms such as abnormal protein aggregation and misfolding, oxidative stress responses, neuroinflammation, and microglial activation (Gadhave et al., 2024). Consequently, current therapeutic strategies for NDs mainly focus on symptomatic treatment. For instance, immunomodulatory therapies are employed in the management of multiple sclerosis (MS), riluzole is used for ALS, and acetylcholinesterase inhibitors are administered for AD. While these medications may have some efficacy in slowing the progression of NDs, their overall therapeutic effects remain suboptima (Bib, 2005). Furthermore, challenges such as poor blood-brain barrier (BBB) permeability and various side effects must also be addressed (Gadhave et al., 2024).

Mitochondrial quality control (MQC) is a critical mechanism for maintaining and regulating the morphology, function, and lifespan of mitochondria. It involves the regulation of various biological processes, including mitochondrial autophagy, biogenesis, dynamics, and genetics (Lin et al., 2023; Roca-Portoles and Tait, 2021). Mitochondrial dysfunction is one of the characteristics of neuronal degeneration, which can directly or indirectly lead to low adenosine triphosphate (ATP) availability, prominent dysfunction, calcium homeostasis and cytoskeletal dynamics disorders, and such changes can cause progressive neuronal death (Plascencia-Villa and Perry, 2023). In addition, the results of genetic studies have elucidated a close correlation between mitochondrial dysfunction and the pathogenesis of NDs (Dupuis, 2014). Targeting MQC to improve mitochondrial dysregulation may represent an effective strategy for the future treatment of NDs (Wang et al., 2021). Although mitochondria play a significant role in NDs, there are currently no approved therapeutic drugs that directly target mitochondria.

Natural products primarily refer to a diverse array of endogenous chemical compounds and metabolites extracted from plants, insects, microorganisms, animals, and the human body (Xing et al., 2023). Natural products have been recognized as a primary target in the development of new pharmaceuticals. Natural products can achieve synergistic regulation through multiple pathways and targets under certain pathological conditions, thereby compensating for the limitations of traditional drug molecules that typically target a single site. Moreover, studies have already found that natural products can serve as alternative therapies for age-related neurological diseases by regulating mitochondrial biogenesis, mitochondrial dynamics, mitochondrial energy metabolism, mitochondrial calcium homeostasis, and mitochondrial oxidative stress (Liang et al., 2021). Therefore, exploring natural products for lead compounds may represent one of the significant approaches in the structural optimization of modern medicinal chemistry (Ma et al., 2022).

Therefore, this review summarizes the functional characteristics of MQC systems and their biological events in the pathogenesis of NDs. Furthermore, we provide a comprehensive summary and analysis of the pharmacological mechanisms by which natural products regulate MQC to improve NDs. This aims to offer a scientific basis for further fundamental research and the development of new clinical drugs.

## 2 Materials and methods

**FIGURE 1:**
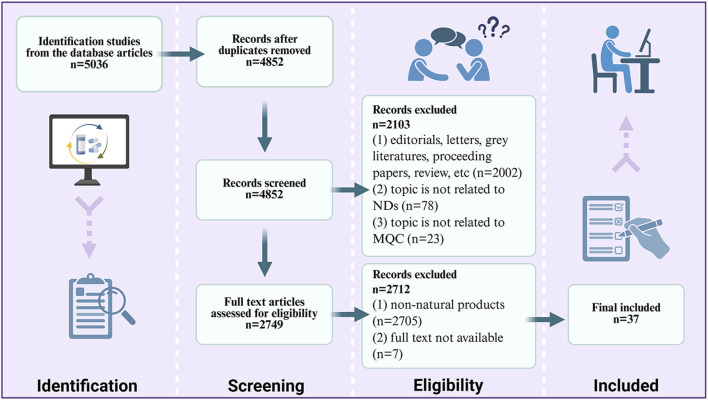
Flow diagram showing the inclusion and exclusion criteria used in the literature.

This review adhered to the Preferred Reporting Items for Systematic Reviews and Meta-Analyses (PRISMA) guidelines and incorporated pertinent literature. A systematic search was conducted utilizing various databases, including Web of Science, PubMed, EMBASE, and MEDLINE. The search strategy employed individual or combined keywords, including mitochondrial biogenesis, mitophagy, mitochondrial dynamics, mitochondria-dependent apoptosis, mitochondrial calcium homeostasis, mitochondrial genetics, mitochondrial oxidative stress, mitochondrial energy metabolism, mitochondrial quality control, Alzheimer’s disease, Parkinson’s disease, Huntington’s disease, and amyotrophic lateral sclerosis. Relevant literatures published up to July 2025 was retrieved. The selection process involved a comprehensive review of titles, abstracts, keywords, and full texts of the articles. The inclusion criteria consisted of original research articles and clinical trials that focused on the regulation of mitochondrial quality control by natural products in the context of neurodegenerative diseases. Exclusion criteria included: (a) editorials; letters; grey literature; conference proceedings; reviews; and duplicate publications; (b) literatures unrelated to the topic or search terms relevant to this study; (c) literatures whose full text could not be obtained. After applying these inclusion and exclusion criteria rigorously, a total of 37 articles with novel findings and significant research relevance were selected for review (Figure 1).

## 3 Pathological characteristics of NDs

The onset of NDs primarily involves alterations in multiple pathological features, including the aggregation of pathogenic proteins, dysfunctions in synaptic and neuronal network activities, abnormalities in protein homeostasis, disruptions in the cytoskeleton, changes in energy metabolism, genetic defects leading to neuronal cell death, among others. These pathological deficiencies combine to collectively drive the process of neurodegeneration (Wilson et al., 2023). AD is pathologically characterized by two prominent lesions: the excessive accumulation of amyloid-β (Aβ) and the hyperphosphorylation of microtubule-associated protein Tau hyperphosphorylated microtubule-associated proteins (Tau), both of which contribute to neuronal dysfunction and loss (Wang et al., 2020). However, the pathological alterations associated with Aβ may be related to synaptic dysfunction, cytoskeletal abnormalities, and disruptions in energy homeostasis (Gao et al., 2024b). The pathological characteristics of PD primarily involve the abnormal accumulation of alpha-synuclein (α-Syn) protein within neurons. This accumulation can trigger neuronal cell death and disrupt dopamine levels, leading to a variety of behavioral and motor disorders (So et al., 2024). The neurodegenerative changes associated with ALS may be linked to mutations in genes such as superoxide dismutase (SOD)1 and TAR DNA-binding protein (TARDBP). These genetic alterations could further induce pathological changes, including neuroinflammation and protein misfolding (Yang and Lee, 2025). HD is an autosomal dominant hereditary neurodegenerative disorder characterized by a progressive worsening of symptoms. It is primarily caused by the pathological expansion of CAG repeat sequences in exon 1 of the huntingtin gene (HTT). An increase in the number of CAG repeats correlates with a progressive exacerbation of neuronal dysfunction and apoptosis, which in turn induces marked atrophy in various regions of the brain (Kim et al., 2021).

## 4 MQC system overview

**FIGURE 2:**
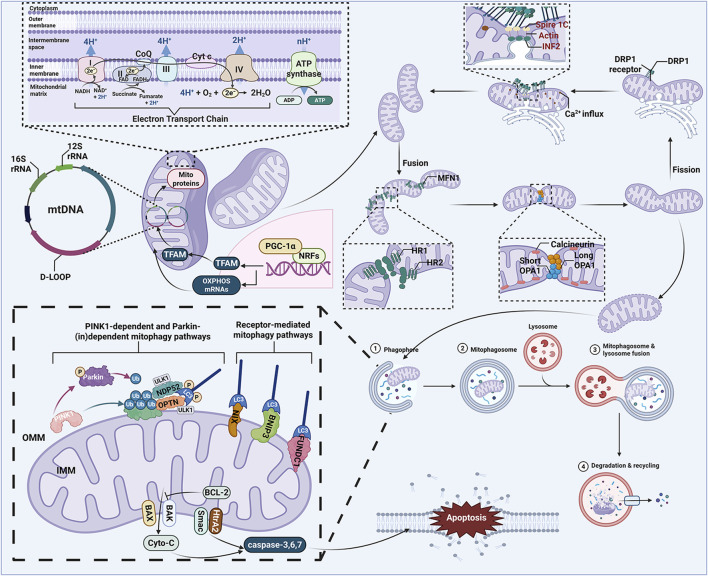
MQC system. The MQC system plays an important role in cellular homeostasis, which mainly involves mitochondrial biogenesis, mitophagy, mitochondrial dynamics, mitochondria-dependent apoptosis mitochondrial calcium homeostasis, mitochondrial genetics, mitochondrial oxidative stress, and mitochondrial energy metabolism. ADP, adenosine diphosphate; ATP, adenosine triphosphate; BAK, BCL-2 associated K protein; BAX, BCL-2 associated X protein; BCL-2, B cell lymphoma 2; BNIP3, BCL2 interacting protein 3; CoQ, ubiquinone; Cyto-C, cytochrome c; D-LOOP, displacement loop; DRP1, dynamin related protein 1; FAD, flavin adenine dinucleotide; FADH, flavine adenine dinucleotide, reduced; FUNDC1, FUN14 domain-containing protein 1; IMM, inner mitochondrial membrane; LC3, light chain 3; MFN1, mitofusin 1; mtDNA, mitochondrial DNA; NADH, nicotinamide adenine dinucleotide; NDP52, Nuclear dot protein 52; NIX, NIP3-like protein X; NRFs, nuclear respiratory factors; OMM, outer mitochondrial membrane; OPA1, optic atrophy protein 1; OPTN, optineurin; OXPHOS, oxidative phosphorylation; PGC-1α, peroxisome proliferator-activated receptor-γ coactivator-1α; PINK1, PTEN-induced putative kinase 1; Smac, second mitochondria-derived activator of caspases; TFAM, mitochondrial transcription factor A; Ub, ubiquitin; ULK1, Unc-51-like kinase 1.

Mitochondria are typically described as small tubular or oval structures (length ∼1–5 μm; width ∼0.5–1 μm) composed of a highly dynamic double membrane, consisting of the outer mitochondrial membrane (OMM) and inner mitochondrial membrane (IMM) (Iovine et al., 2021). As the “powerhouse” and metabolic hub of eukaryotic cells, mitochondria primarily function to produce ATP through the breakdown of carbohydrates and fatty acids *via* oxidative phosphorylation (OXPHOS), providing energy for cellular development. This process relies on the support of the electron transport chain (ETC) located on the IMM. During this process, proton pumps (Complexes I, III, and IV) transfer protons from the mitochondrial matrix to the intermembrane space (IMS), creating a proton motive force and membrane potential (ΔΨm) essential for ATP production (An et al., 2021). Under physiological conditions, mitochondria display a high degree of plasticity—adapting their morphology, structure, function, and even quantity dynamically in response to fluctuations in cellular physiological activities and external environmental stimuli to maintain metabolic homeostasis (Ni et al., 2014). This is critical for maintaining mitochondrial homeostasis and cellular adaptability. However, mitochondrial dysfunction can lead to ATP deficiency, overload of superoxide anions, increased levels of pro-apoptotic molecules, and ultimately result in cell death (Yan et al., 2020). Therefore, as an intrinsic cellular protective mechanism, the MQC system is crucial for improving mitochondrial defects and maintaining mitochondrial function under stress conditions (Pickles et al., 2018). It encompasses various physiological and biochemical processes, including mitophagy, energy metabolism, biogenesis, and oxidative stress (Jiang et al., 2024; Song et al., 2023). The dysregulation or damage of the MQC system can lead to various mitochondrial dysfunctions, which may trigger multiple diseases such as chronic obstructive pulmonary disease, cardiovascular disorders, neurodegenerative diseases, cancer, and diabetes (Liu Y.-B. et al., 2024). Recent studies further imply that changes in mitochondrial biogenesis and mitophagy are closely associated with the pathological alterations of neurodegenerative diseases (Wang et al., 2019) (Figure 2).

## 5 MQC system in NDs: their role and significance

### 5.1 Mitochondrial biogenesis

Mitochondrial biogenesis is a complex process through which mitochondria achieve self-renewal. This process requires the coordinated expression and regulation of over 1,100 proteins encoded by both nuclear and mitochondrial genomes (Gureev et al., 2019; Safdar et al., 2018). Peroxisome proliferator-activated receptor-γ coactivator-1α (PGC-1α) is considered a key regulatory factor in mitochondrial biogenesis, as it plays a crucial role in promoting the expression of numerous other genes and the assembly of proteins (Gureev et al., 2019). The activation of PGC-1α initiates the activation of nuclear respiratory factor 1 and 2 (Nrf1 and Nrf2), which subsequently promotes the transcription of mitochondrial genes, including those encoding components of the ETC., such as ATP synthase and mitochondrial transcription factor A (TFAM). TFAM is then translocated to the mitochondrial matrix, where it stimulates mitochondrial DNA (mtDNA) replication and enhances mitochondrial gene expression (Simmons et al., 2020). Moreover, the activation of PGC-1α not only mediates the expression of mtRNA for reactive oxygen species (ROS) detoxifying enzymes but also promotes the expression of OXPHOS subunits to maintain mitochondrial respiratory chain functionality (Cardanho-Ramos and Morais, 2021). During the process of mitochondrial biogenesis, several cellular signaling pathways are closely associated with the PGC-1α-Nrf1/2-TFAM-mediated mitochondrial biogenesis. The adenosine monophosphate (AMP)-PGC-1α axis and the sirtuin 1 (SIRT1)-PGC-1α axis are two primary pathways regulating mitochondrial biogenesis (Delghandi et al., 2005; Gerhart-Hines et al., 2007; Jäger and Handschin, 2007). Ca^2+^ is also a crucial regulatory factor in mitochondrial biogenesis. On one hand, Ca^2+^ activates the calmodulin-dependent protein kinase (CaMK)/p38 mitogen-activated protein kinase (MAPK) pathway, leading to the activation and expression of PGC-1α. On the other hand, it has been demonstrated that CaMK can stimulate PGC-1α activation by mediating the expression of cAMP response element-binding protein (CREB), ultimately resulting in an increase in mitochondrial biogenesis (Cardanho-Ramos and Morais, 2021).

Clinical research has established the relationship between mitochondrial biogenesis and the pathophysiological mechanisms underlying several common NDs. Specifically, previous studies have reported reduced expression levels of PGC-1α—a central regulator of mitochondrial biogenesis—in the cortical nuclei of patients with AD. The overexpression of PGC-1α in N2a neuroblastoma cells has been shown to decrease Aβ levels (Katsouri et al., 2011). Furthermore, the expression of mtDNA OXPHOS genes is also diminished in brain samples from AD patients, which may negatively impact the normal functioning of the mitochondrial respiratory chain and ATP production (Bennett and Keeney, 2020). Recent clinical studies have observed a reduction in the levels of PGC-1α in brain tissue from patients with PD post-mortem, which may reflect impairments in mitochondrial biogenesis (Soyal et al., 2019). In order to assess the changes in mitochondrial biogenesis and content, clinical studies have revealed a significant reduction in the expression levels of PGC-1α and TFAM in peripheral mononuclear cells of patients with ALS (Araujo, 2020). Clinical research has found that common polymorphisms and prevalent haplotypes at the PPARGC1A locus (which encodes PGC-1α) influence the age of onset of HD. This provides crucial support for the role of PGC-1α-mediated mitochondrial biogenesis in human HD pathogenesis (Weydt et al., 2009).

The role of mitochondrial biogenesis in various models of NDs has also been demonstrated through animal experiments. Animal studies have also revealed a reduction in the levels of PGC-1α, Nrf1, Nrf2, and TFAM in the brain tissue of triple transgenic Alzheimer’s disease mouse models (3 × Tg-AD) (Singulani et al., 2020). Furthermore, targeting and regulating PGC-1α to induce mitochondrial biogenesis has been shown to prevent and ameliorate learning deficits and hippocampal degeneration in AD mouse models (Jamwal et al., 2021). Notably, in PD, activation of the SIRT1/PGC-1α pathway has been mechanistically linked to disease pathogenesis. Experimental evidence reveals that targeted enhancement of the SIRT1/PGC-1α/TFAM axis increases tyrosine hydroxylase (TH)-positive neurons in the substantia nigra pars compacta (SNpc), elevates BDNF expression, and suppresses neuroinflammatory mediators in murine models (Hsu et al., 2022). Besides, targeting AMPK/PGC-1α-mediated mitochondrial biogenesis may alleviate motor deficits and degeneration of dopaminergic neurons in PD model mice (Cheng et al., 2021). Current research on ALS-related mechanisms has revealed that targeted upregulation of PGC-1α-mediated mitochondrial biogenesis in animal models significantly attenuates skeletal muscle atrophy in ALS pathophysiology. Furthermore, the resultant increase in ATP production has been shown to enhance muscle endurance (Da Cruz et al., 2012). In recent years, multiple animal studies have demonstrated the positive role of PGC-1α targeting mitochondrial biogenesis in ameliorating neuronal degeneration in HD models (Di Cristo et al., 2019; Jesse et al., 2017). This process also enhances ATP supply for cellular energy. The aforementioned evidence suggests that impairments in mitochondrial biogenesis may be one of the potential pathological factors common to neurodegenerative diseases.

### 5.2 Mitophagy

Autophagy is an evolutionarily conserved intracellular degradation process in which cytoplasmic substrates are engulfed by autophagosomes that subsequently fuse with lysosomes for degradation and recycling. This mechanism plays a crucial role in maintaining cellular nutrient homeostasis and organelle quality control (Levine and Kroemer, 2019). Mitophagy refers to the process by which cells selectively isolate and degrade defective mitochondrial components through autophagy. This mechanism is crucial for maintaining mitochondrial integrity and metabolic homeostasis, serving as one of the key pathways for mitochondrial quality control (Cai and Jeong, 2020). Mitophagy primarily encompasses two regulatory pathways: the ubiquitin-dependent mitophagy pathway and the non-ubiquitin-dependent mitophagy pathway. The ubiquitin-dependent mitophagy is predominantly mediated by the PTEN-induced putative kinase 1 (PINK1)-parkin pathway, which is a highly conserved mitochondrial protein. PINK1 plays a crucial role in regulating mitochondrial function; under normal circumstances, PINK1 exists within cells in an auto-inhibitory form (Wauer and Komander, 2013). Mitochondrial damage or depolarization can modify parkin and activate its E3 ubiquitin ligase activity. PINK1 accumulates on the outer mitochondrial membrane, initiating the activation process of parkin, which involves phosphorylation, multiple conformational changes, and association with Ser65-phosphorylated ubiquitin. This cascade ultimately leads to the ubiquitination of proteins on the outer mitochondrial membrane (Harper et al., 2018). Autophagy receptors, such as optineurin (OPTN), p62, and calcium binding and coiled-coil domain 2 (NDP52), specifically interact with ubiquitin proteins (Zeng et al., 2022). These autophagy receptors facilitate the formation of autophagosomes by binding to light chain 3 (LC3) through their interaction motifs, thereby initiating the mitophagy process (Guan et al., 2018). In addition to their role in ubiquitin-dependent mitophagy, a subset of mitochondrial proteins also functions as mitophagy receptors, directly targeting defective mitochondria for isolation and degradation within autophagosomes (Palikaras et al., 2018). This process is mediated by several proteins, including FUN14 domain-containing protein 1 (FUNDC1), NIP3-like protein X (NIX), and B cell lymphoma 2 (BCL-2) interacting protein 3 (BNIP3) (Liu et al., 2012; Schweers et al., 2007; Zhang et al., 2016).

Clinical research has revealed a relationship between mitophagy-related proteins and common NDs. In patients with AD, the levels of PINK1 are abnormally elevated in both cerebrospinal fluid and peripheral blood. Furthermore, transcription factor EB (TFEB), which serves as a positive regulatory factor for autophagy and lysosomal biogenesis, is found to be reduced in both cerebrospinal fluid and peripheral blood (Veverová et al., 2024; Zhang et al., 2024). In addition, research has identified that PINK1 mediates the clearance of Aβ by enhancing parkin signaling pathways, which holds significant potential for improving neuronal degeneration in AD (Martín-Maestro et al., 2016). The loss of function of PINK1 or parkin is associated with the onset of autosomal recessive hereditary PD. Understanding the roles of PINK1 and parkin in PD is crucial for advancing our knowledge in this field (Malpartida et al., 2021). Research has shown that the mitophagy pathway involving PINK1/parkin is impaired in the brain tissue of patients with PD (Zilocchi et al., 2018). Furthermore, the specific expression of mitophagy-related proteins (PINK1 and parkin) in the plasma of PD patients may serve as distinctive biomarkers for diagnosing PD (Qian et al., 2023). In the study of ALS, previous research has clearly demonstrated through electron microscopy that pathological mitophagy produces distinct ultrastructural effects within specific regions of motor neurons (Ruffoli et al., 2015). Research has shown that the expression of mitophagy proteins is abnormal in peripheral mononuclear cells of ALS patients, characterized by the upregulation of PINK1 and LC3Ⅱ(Bordoni et al., 2022). Clinical studies on the connection between mitophagy and HD are currently limited, but some research has found that the interaction between autophagy receptors and autophagosomes is disrupted in HD patients’ brain tissue. This impairment blocks the transfer of ubiquitin-tagged damaged mitochondria to autophagosomes, impairing mitophagy (Martinez–Vicente et al., 2010).

In both *in vivo* and *in vitro* experimental studies, research has indicated a region-specific correlation between the abnormal alterations of mitophagy and the neuropathology associated with AD. Furthermore, these findings suggest that mitophagy could serve as a potential neuroprotective pathway (Hou et al., 2021). Research has shown that the targeted modulation of the PINK1/parkin pathway to induce mitophagy can ameliorate Tau protein phosphorylation and excessive accumulation of Aβ in AD models. This intervention subsequently leads to improvements in memory deficits and neuronal damage observed in these models (Fang et al., 2019). PARIS (ZNF746), as a pathological substrate of parkin, is found to be increased in relevant brain regions associated with both sporadic and familial PD (Shin et al., 2011). Interestingly, research has shown that the inhibition of PARIS can not only ameliorate mitophagy dysfunction caused by mutations in parkin or PINK1 but also sustain PGC-1α-mediated mitochondrial biogenesis. This dual action may be beneficial for neuronal degeneration associated with PD (Lee et al., 2017). FUNDC1 plays a critical role in the apoptosis of spinal cord neurons in ALS models. Research has shown that FUNDC1-dependent mitophagy can improve mitochondrial health in motor neurons of ALS model mice and reduce neuronal apoptosis, thereby extending the lifespan of SOD1 mutant mice (Guo et al., 2024). Mutant huntingtin protein (mHtt) alters multiple cellular processes, leading to neuronal dysfunction and cell death. Research has demonstrated that the presence of mHtt reduces mitochondrial targeting by autophagosomes, resulting in an imbalance between mitochondrial accumulation and mitophagic clearance in striatal neurons. However, upregulating PINK1-mediated mitophagy can prevent mHtt-induced neurotoxicity (Khalil et al., 2015).

### 5.3 Mitochondrial dynamics

In normal physiological conditions, mitochondria are dynamic organelles that continually undergo morphological changes to maintain mitochondrial homeostasis and adapt to cellular energy demands. The dynamic morphological alterations of mitochondria include processes of fusion and fission, which facilitate the adequate distribution of mitochondria within the cell (Rovira-Llopis et al., 2017). Several guanosine triphosphate hydrolases (GTPases) associated with dynamin proteins constitute the core mechanism underlying mitochondrial fusion and fission processes. Mitofusin 1 and 2 (Mfn1/2) act synergistically by promoting the formation of both homotypic and heterotypic oligomers, thereby inducing the fusion of the OMM. This process establishes network communication between mitochondria and maintains their integrity (Grel et al., 2023). Additionally, optic atrophy protein 1 (OPA1) is located in the inner mitochondrial membrane and intermembrane space, primarily responsible for the fusion of the IMM and the remodeling of mitochondrial cristae structures. Long-OPA1 (L-OPA1) and short-OPA1 (S-OPA1) are two isoforms of OPA1, with their relative levels being key determinants of mitochondrial fusion activity (Chen W. et al., 2023). The proteins involved in mitochondrial fission include dynamin related protein 1 (Drp1) and mitochondrial fission 1 protein (FIS1). The recruitment of Drp1 involves various OMM adaptor proteins, including FIS1, mitochondrial fission factor (Mff), and the mitochondrial dynamics proteins MiD-49 and MiD-51. Drp1 is recruited to the OMM in a highly oligomerized helical structure, which enhances the GTPase activity at the sites of mitochondrial fission. This process subsequently drives membrane constriction, ultimately leading to the division of mitochondria (Flippo and Strack, 2017). Mitochondrial fusion and fission dynamics play a crucial role in regulating the morphology of mitochondria, thereby maintaining their normal functions at both physiological and pathological levels. These processes are significant for facilitating the exchange of mitochondrial contents and for the removal of damaged organelles (Liu B.-H. et al., 2024).

In clinical research, some investigators have analyzed the expression patterns of mitochondrial fission and fusion proteins in hippocampal tissue from AD patients. The results indicated that the levels of Drp1, OPA1, Mfn1, and Mfn2 were significantly reduced in the hippocampus of AD patients, which is consistent with the abnormal changes in mitochondrial dynamics observed in AD (Wang et al., 2009). Research has shown that mitochondrial damage is present in the substantia nigra specimens of patients with PD. Among these findings, mitochondrial fusion impairment due to reduced levels of L-OPA1 and S-OPA1 is a common occurrence (Zilocchi et al., 2018). Although physiological fission is essential for maintaining mitochondrial quality, excessive mitochondrial fragmentation mediated by Drp1 can lead to adverse events such as increased mitochondrial fragmentation, depolarization of the mitochondrial membrane, and accumulation of ROS (Shirihai et al., 2015). Therefore, clinical studies have found that blocking the interaction between Drp1 and FIS1 can reduce the pathological progression of ALS (Joshi et al., 2018). Additionally, research has indicated that in the brains of patients with Huntington’s disease (HD) postmortem, there is an increased expression of Drp1 and FIS1, while the expressions of Mfn1, Mfn2, and OPA1 are reduced. This suggests the presence of abnormal mitochondrial dynamics (Shirendeb et al., 2011).

In both *in vivo* and *in vitro* experimental studies, researchers utilizing AD models have identified a correlation between mitochondrial dynamics impairment and the excessive production of Aβ peptides. In the brains of AD model mice, levels of Mfn1, Mfn2, and L-OPA1 are found to be reduced, while the level of the fission protein DRP1 is elevated. This imbalance in mitochondrial dynamic proteins represents an early event in the pathological progression of AD (de la Cueva et al., 2022). Research has shown that mitochondrial dynamics impairment induced by DJ-1 mutations related to PD is associated with the condition. In mouse models lacking DJ-1, there is an overexpression of Drp1 in specific brain regions, which leads to a failure of mitochondrial fusion. This may result in dopaminergic deficits in the substantia nigra-striatum pathway and subsequently cause a decline in motor function in these mice (Wang et al., 2012). In the ALS *Drosophila* model, mitochondrial dynamics exhibit an imbalance characterized by increased mitochondrial fission. However, co-regulation of the genes OPA1, Mfn, and Drp1 can rescue the fragmentation of mitochondrial morphology induced by ALS in fruit flies (Altanbyek et al., 2016). Furthermore, similar pathological changes have been observed in striatal neural progenitor cells within HD models, where there is an increase in the expression of Drp1 and FIS1 alongside a decrease in Mfn1, Mfn2, and OPA1. Restoring the balance between mitochondrial fission and fusion has been shown to reduce mitochondrial damage and protect synapses in neurons from HD models, highlighting its therapeutic potential for targeting HD-related neuronal impairment (Manczak and Reddy, 2015).

### 5.4 Mitochondria-dependent apoptosis

One of the critical roles of mitochondria is to induce apoptosis. Mitochondria-dependent apoptosis represents one of the primary endogenous pathways for programmed cell death, which can be triggered by various factors such as oxidative stress, DNA damage, and oncogene activation (Green, 2022). These changes can induce alterations in the mitochondrial membrane structure, thereby promoting the release of pro-apoptotic proteins such as cytochrome c (Cyto-C), Smac/Diablo, and HtrA2/Omi, which are normally sequestered within the mitochondria, into the cytosol (Li et al., 2023). Concurrently, these alterations may lead to a decrease in ΔΨm, respiratory deficiencies, and an increase in ROS production, all of which contribute to mitochondrial dysfunction (Bossy-Wetzel et al., 2003). Mitochondria-dependent apoptosis is regulated by the BCL-2 protein family, which comprises anti-apoptotic proteins (BCL-2-like proteins), pro-apoptotic proteins such as BCL-2 associated X protein (BAX) and BCL-2 associated K protein (BAK), as well as BH3-only proteins (Cory and Adams, 2002). The BAX/BAK proteins directly participate in apoptosis by regulating the permeability of the OMM. Specifically, once Cyto-C is released after recognition by BAX/BAK proteins, it binds to apoptotic protease activating factor-1 (Apaf-1), promoting its oligomerization. This process subsequently facilitates the activation of caspase-9 and the assembly of apoptosomes. Following this, these apoptosomes activate caspases 3, 6, and 7, ultimately leading to cell apoptosis (Yuan and Ofengeim, 2024). Additionally, Smac/Diablo and HtrA2/Omi promote the activation of caspase-3, -6, and -7 by inhibiting inhibitors of apoptosis proteins (IAPs), thereby facilitating apoptosis (Tower, 2015). Some apoptosis-inducing proteins that do not rely on Caspases, such as apoptosis-inducing factor (AIF) and endonuclease G (Endo G), can translocate from the mitochondria to the nucleus. This translocation leads to DNA fragmentation and chromatin condensation, ultimately resulting in cell apoptosis (Joza et al., 2001).

The clinical research regarding the correlation between mitochondria-dependent apoptosis and neurodegenerative diseases remains insufficient. Caspase-7 is a direct target of miR-335-5p. Clinical studies have shown that the downregulation of miR-335-5p in the serum of patients with ALS enhances mitochondrial dynamics disturbance and neuronal apoptosis (De Luna et al., 2020). Additionally, clinical studies have shown that cybrids from HD patients undergo apoptosis induced by 3-nitropropionic acid (3-NP), which involves the release of Cyto-C and mitochondrial AIF. This process is associated with the translocation of mitochondrial BAX (Ferreira et al., 2010). In contrast, numerous preclinical studies have identified the critical regulatory role of mitochondrial-dependent apoptosis in several common neurodegenerative diseases. As a known E3 ubiquitin ligase, the transient silencing of p53-induced RING-H2 protein (Pirh2) has been shown to reduce levels of pathological markers characteristic of AD, such as hyperphosphorylation of Tau protein and excessive accumulation of Aβ. These alterations are closely associated with the inhibition of cytochrome c release and its ubiquitination-mediated neuronal apoptosis. Furthermore, silencing Pirh2 also suppresses the translocation of AIF and Endo G, thereby alleviating DNA damage (Singh et al., 2024)。Rapamycin is a lipophilic macrolide antibiotic that is widely utilized as an immunosuppressant in clinical settings (Noda and Ohsumi, 1998). Recent studies have demonstrated that it can reduce the activation of caspase-9 by decreasing the release of Cyto-C from the intermembrane space of mitochondria. This effect may attenuate oxidative stress and apoptotic responses in neurons within PD models (Jiang et al., 2013). Apaf-1 and apoptosomes play crucial roles in the induction of apoptosis. Research has shown that even in the context of Cyto-C release, inhibiting the Apaf-1-mediated caspase cascade can prevent mitochondrial damage and cell death, thereby improving neurodegeneration in ALS cellular models (Cozzolino et al., 2006). Minocycline is a semi-synthetic tetracycline that has been shown to possess extensive neuroprotective properties in experimental models. Research indicates that minocycline can improve the degeneration and apoptosis of striatal neurons in a HD mouse model by inhibiting both caspase-independent and caspase-dependent apoptotic pathways (Wang et al., 2003).

### 5.5 Mitochondrial calcium homeostasis

In nearly all eukaryotic cells, mitochondria function as the primary organelles responsible for mediating intracellular Ca^2+^ signaling. They are capable of buffering Ca^2+^ by coordinating uptake and efflux pathways, thereby establishing a spatiotemporal homeostasis of Ca^2+^ within the cytoplasm (Arduino and Perocchi, 2018). Ca^2+^ can enter the mitochondria through the mitochondrial Ca^2+^ uniporter (MCU) and voltage-dependent anion channels (VDACs) located in the outer mitochondrial membrane. The release of Ca^2+^ from the mitochondria occurs primarily *via* three mechanisms: Na^+^-Ca^2+^ exchanger, H^+^-Ca^2+^ exchanger, and the mitochondrial permeability transition pore (mPTP) (Zhao et al., 2022). It is crucial to note that the imbalance of mitochondrial calcium homeostasis serves as a sensitizing signal for apoptosis. Excessive accumulation of Ca^2+^ within the mitochondria can induce mitochondrial swelling and even rupture, thereby facilitating the release of pro-apoptotic proteins from the mitochondria (Giorgi et al., 2012).

In clinical research, some investigators have found that mitochondria isolated from fibroblasts of AD patients exhibit a reduced capacity for Ca^2+^ uptake. This finding suggests that the Ca^2+^ buffering ability of mitochondria in AD fibroblasts may be compromised (Kumar et al., 1994). Moreover, mitochondria extracted from the brain tissue of AD patients with overexpression of amyloid precursor protein (APP) also exhibited a reduced Ca^2+^ capacity (Du et al., 2008). The mitochondrial Rho GTPase Miro1 plays a critical role in maintaining mitochondrial calcium homeostasis (Saotome et al., 2008). In fibroblasts derived from PD patients with mutations in Miro1, there is an increase in cytosolic calcium levels and a reduction in mitochondrial uptake, indicating impaired mitochondrial calcium homeostasis (Grossmann et al., 2019). Additionally, research has shown that the levels of free Ca^2+^ in peripheral blood lymphocytes of sporadic ALS patients are elevated in a resting state. These findings may be attributable to mitochondrial dysfunction or variations in the content of calcium-binding proteins within the affected motor neurons (Curti et al., 1996). Research has shown that the mitochondrial calcium capacity in lymphoblasts from patients with HD is significantly lower than that of the control group. This reduction in calcium load induces mitochondrial depolarization through the mPTP, ultimately leading to cell death (Panov et al., 1999).

Research has shown that the proper uptake and efflux of mitochondrial Ca^2+^ are crucial for maintaining mitochondrial calcium homeostasis. Under pathological conditions associated with AD, it was found that the accumulation of Aβ oligomers promotes mitochondrial Ca^2+^ influx through MCU, leading to mitochondrial Ca^2+^ overload and the activation of pro-apoptotic factors, ultimately resulting in neuronal cell death (Calvo-Rodriguez et al., 2020). Mitochondrial Ca^2+^ uptake is excessively increased, which can induce pathological changes in various neurodegenerative disease models, including PD (Twyning et al., 2024). For instance, in PD models with PINK1 mutations involving dopaminergic neurons, the activity of the endoplasmic reticulum (ER)-mitochondria contact sites (ERMCSs) is enhanced. This results in elevated mitochondrial Ca^2+^ levels, leading to an increase in mitochondrial size and neuronal death (Lee K.-S. et al., 2018). Calcium excitotoxicity is a critical trigger factor in the etiology of ALS. Research has indicated that reduced Ca^2+^ uptake in the mitochondrial matrix of ALS cellular models may lead to the release of Ca^2+^ into the cytoplasm. This process causes calcium excitotoxicity and excessive accumulation of ROS, ultimately resulting in motor neuron death (Jaiswal, 2017). Furthermore, studies have shown that the disruption of the ER–mitochondrial network in the striatum of HD models negatively impacts the buffering capacity of mitochondria for Ca^2+^ and results in functional impairment and degeneration of the striatal tissue (Cherubini et al., 2020).

### 5.6 Mitochondrial genetics

Unlike other organelles, mitochondria are inherited maternally and possess an independent genetic system as well as a protein translation system. The mtDNA consists of a circular DNA molecule containing 16,568 base pairs, which includes one light chain and one heavy chain. It encompasses 37 genes, comprising 13 polypeptides involved in encoding transcription and translation, 22 transfer RNAs(tRNAs), 2 ribosomal RNAs (rRNAs), and a displacement loop (D-loop) (Shukla and Mukherjee, 2020). The 13 polypeptides encoded by mtDNA are integral components of the subunits of mitochondrial respiratory chain complex I-V, playing a crucial role in ATP production through OXPHOS (Schon et al., 2012). The D-loop is a non-coding region within mtDNA that encompasses regulatory elements essential for mtDNA replication and transcription (Sharma et al., 2005). mtDNA is the sole source of essential cellular proteins outside the eukaryotic cell nucleus. In comparison to nuclear DNA, mtDNA lacks protective histone wrapping and has limited repair mechanisms (Dunham-Snary and Ballinger, 2013). The integrity of mtDNA is continuously compromised by ROS generated during cellular OXPHOS processes (Wiggs, 2015). Furthermore, the mutation rate of mtDNA is nearly ten times higher than that of nuclear DNA (Brown et al., 1979). The proximity of mtDNA to ROS production sites within mitochondria serves as a primary cause of mtDNA instability (Chinnery and Hudson, 2013). The extensive range of mitochondrial gene mutation disorders indicates that mitochondrial dysfunction can lead to a variety of clinical phenotypes associated with metabolic and degenerative diseases, as well as cancer and aging. The symptoms of mitochondrial diseases may be partly attributed to variations in the mitochondrial genome (Wallace and Fan, 2009).

Clinical research has shown that mtDNA disturbances play a role in the pathogenesis of various neurodegenerative diseases. In the neurons of patients with AD, there is an observed increase in mtDNA fragmentation and a decrease in mtDNA content, leading to impaired mitochondrial function and heightened susceptibility of neurons to oxidative damage (de la Monte et al., 2000). In addition, a study has found that in the brain tissue of patients with PD after death, there is a specific accumulation of mtDNA damage in certain brain regions, which parallels an increase in mtDNA damage in peripheral tissues. In future clinical trials, mtDNA damage may serve as one of the blood biomarkers for PD (Qi et al., 2023). In clinical studies of ALS, compared to non-carriers of ALS-related gene mutations (*SOD1*, *TARDBP*, *FUS*, and *C9orf72*), ALS patients exhibit a significant increase in mtDNA copy number. Additionally, there appears to be a trend toward decreased D-loop methylation levels. In contrast, these two biomarkers are observed at moderate levels in asymptomatic/symptomatic carriers (Stoccoro et al., 2018). Additionally, research has shown that the instability of the Htt CAG repeat sequence within human cell nuclei leads to a high-level expression of mHtt fragments. This, in turn, affects mitochondrial network dynamics and mitophagy, resulting in pathogenic mtDNA mutations (Neueder et al., 2024).

Mechanistic studies have revealed that the proofreading function of mtDNA polymerase-γ (PolgA D257A) induces an accumulation of mtDNA mutations associated with aging. This accumulation leads to mitochondrial dysfunction and exacerbates the neurodegenerative changes in AD mouse models induced by amyloid pathology (Kukreja et al., 2014). Type I interferon (IFN) β or IFNAR gene deletion-induced PD models in mice may exhibit pathology associated with mtDNA mutations. The absence of endogenous neuronal IFNβ-IFNAR signaling can lead to mtDNA variations and oxidative damage, thereby resulting in the onset and propagation of neurotoxicity (Tresse et al., 2023). Additionally, research has demonstrated that overexpression of mitochondrial TFAM can protect mtDNA from oxidative stress and promote mtDNA transcription, thereby delaying the onset of disease in ALS model mice (Morimoto et al., 2012). In the study of mechanisms underlying HD, a deficiency in melatonin has been shown to exacerbate mitochondrial ROS damage and subsequently lead to the release of mtDNA. This process activates cyclic guanosine monophosphate-adenosine monophosphate synthase (cGAS) and inflammatory pathways in HD model neurons, ultimately resulting in synaptic loss and neurodegeneration (Jauhari et al., 2020).

### 5.7 Mitochondrial oxidative stress

ROS, as byproducts of cellular metabolism, serve as crucial signaling molecules that play significant roles in both physiology and disease. The mitochondrial respiratory chain is the primary source of ROS within cells; during ATP production utilizing oxygen, mitochondria concurrently release ROS (Mansouri et al., 2006). Under normal physiological conditions, mitochondria regulate the accumulation of ROS through a series of intricate antioxidant defense mechanisms within the cell. The buildup of ROS triggers the activation of various antioxidant enzymes, including SOD2, catalase (CAT), glutathione peroxidase/reductase (GSH-PX) system, and peroxiredoxin/thoredoxin (PRX/Trx) system, to prevent oxidative damage to organelles (Lenaz et al., 2002). However, in the context of aging or various disease states, the dynamic balance between mitochondrial oxidative stress and antioxidant defense systems is disrupted, leading to an increased release of ROS. This disruption further exacerbates the pathological changes associated with these diseases (Kowalczyk et al., 2021). The brain, characterized by a high metabolic demand (and thus heavy mitochondrial activity) and limited cell regenerative capacity, is disproportionately susceptible to oxidative injury caused by mtROS relative to other tissues. This biological trait strongly implies that mitochondrial oxidative stress is a critical risk factor contributing to the initiation and progression of neurodegenerative changes (Wen et al., 2025).

Clinical research has revealed that patients with AD exhibit elevated levels of oxidative damage in the mitochondria of peripheral lymphocytes, which may serve as a potential biomarker for AD in the future (Sultana et al., 2011). Furthermore, compared to healthy individuals, sporadic AD patients exhibit elevated levels of mitochondrial ROS in their fibroblasts (Li Y. et al., 2024). Research has shown that the activities of SOD and GSH-PX in the substantia nigra and basal ganglia of PD patients exhibit significant differences compared to healthy individuals. This suggests the potential presence of oxidative toxicity and free radical damage in these brain regions (Choi et al., 2005; Marttila et al., 1988). The pathological changes associated with ALS may be related to mitochondrial-dependent oxidative stress. Research has demonstrated a high degree of heterogeneity in the levels of SOD1, GSH-PX, and oxidative stress between peripheral lymphocytes from ALS patients and those from healthy individuals (Cunha-Oliveira et al., 2022). Furthermore, research has indicated that there is an upregulation of CAT in the mitochondria of fibroblasts from patients with HD. This finding suggests that the neurodegenerative changes associated with HD may be accompanied by oxidative stress damage (del Hoyo et al., 2006).

Research has demonstrated that mitochondrial-mediated oxidative stress induces the formation and accumulation of Tau oligomers. This finding indicates a correlation between mitochondrial oxidative stress and the pathological changes associated with AD. Furthermore, targeting the clearance of mtROS can reduce the accumulation of Tau protein in neurons of AD mouse models (Du et al., 2022). Due to their unique physiological and morphological characteristics, dopaminergic neurons in the SNpc are particularly susceptible to the effects of oxidative stress (Imbriani et al., 2022). Furthermore, studies have indicated that there is an upregulation of mtROS in PD models, and inhibiting mtROS can mitigate the progressive pathological changes observed in dopaminergic neurons (Zhao et al., 2025). The *C9orf72* mutation is the most prevalent pathogenic mutation associated with ALS (DeJesus-Hernandez et al., 2006). Research has shown that targeting the Kelch-1ike ECH- associated protein l (Keap1)/Nrf2 signaling pathway to combat mitochondrial oxidative stress represents a potential therapeutic strategy for *C9orf72*-related ALS (Au et al., 2024). Additionally, research has found mitochondrial oxidative stress in the striatum of HD model rats, along with reduced activity of manganese superoxide dismutase (Mn-SOD) and CAT—key antioxidants protecting mitochondrial function (Mehrotra et al., 2015). The aforementioned evidence suggests that mitochondrial-mediated oxidative stress may contribute to the neurodegenerative pathological changes observed in this condition.

### 5.8 Mitochondrial energy metabolism

As the primary driving force for cellular function, mitochondria directly fulfill most of the energy demands of the organism through the production of ATP. This process is contingent upon OXPHOS and is primarily regulated by four oligomeric protein complexes located in the mitochondrial inner membrane’s ETC., namely Complexes I, II, III, and IV (Jiang et al., 2024). ATP production decline can impair ATP-dependent processes, consequently affecting the functionality of all cells in the body (Hroudová et al., 2016). The mitochondrial energy metabolism process is crucial for maintaining mitochondrial homeostasis. A reduction in mitochondrial energy transduction capacity is a common feature associated with brain aging and neurodegeneration.

Clinical research has revealed that oxidative damage to the mitochondrial ATP synthase α subunit occurs in the entorhinal cortex during Braak stage I/II of AD. This finding suggests a close relationship between early tau protein pathology and alterations in the mitochondrial energy production system in AD (Terni et al., 2009). Recent studies have revealed the presence of latent metabolic impairment in fibroblasts derived from patients with sporadic PD. These fibroblasts exhibit reduced ATP levels, and this effect is exacerbated when OXPHOS sustains further damage (Deus et al., 2020). In the context of ALS, researchers have observed a significant reduction in the expression of key metabolic genes in the peripheral blood of ALS patients. This is primarily characterized by decreased levels of metabolic compounds such as ATP and pyruvate, suggesting that mitochondrial energy metabolism dysregulation may be associated with pathological changes in ALS (Araujo, 2020). Previous studies have found that both HD patients and asymptomatic gene carriers show a significant reduction in ATP synthesis rate compared to healthy individuals. This mitochondrial energy metabolism impairment is likely a component of HD-related pathological injury (Saft et al., 2005).

Mechanistic studies have revealed a positive correlation between the deficiency of ATP in the brain and the accumulation of Aβ in the brains of AD model mice. Furthermore, it has been demonstrated that tridecanoic acid exhibits protective effects against Aβ-mediated cerebral energy deficits and mitochondrial dysfunction. These findings suggest that cerebral energy depletion plays a critical role in the pathogenesis of AD (Yuan et al., 2020). Mitochondrial energy metabolism may play a synergistic role in neurodegenerative changes. Research has indicated that nanoplastics could promote neurodegenerative lesions in PD mice by inducing disturbances in energy metabolism within the substantia nigra and striatum (Liang et al., 2022). The dysfunction of bioenergetics may be associated with motor neuron diseases. Recent studies have discovered that the synaptic defects in motor neurons of ALS fruit flies carrying the *vapb* ^*P58S*^ mutation could result from a decoupling between mitochondrial ATP production and neuronal activity consumption (Karagas et al., 2022). Furthermore, research has indicated that the aggregation of mHtt in HD interacts with the mitochondrial energy metabolism defects induced by equilibrative nucleoside transporter (ENT)2, which further exacerbates the pathological damage observed in HD mice (Chen C.-Y. et al., 2023).

## 6 Natural products in the prevention and treatment of NDs through mitigating mitochondrial dysfunction

### 6.1 Improving mitochondrial biogenesis

Baicalein is one of the flavonoid compounds isolated from the root tissue of the traditional Chinese herb *Scutellaria baicalensis* Georgi. It has shown potential in treating various conditions, including inflammation, hypertension, and cardiovascular diseases. Previous studies have demonstrated that baicalein can inhibit the degeneration of dopaminergic neurons (Mu et al., 2009). Furthermore, research has shown that it can enhance mitochondrial biogenesis by activating the CREB/glycogen synthase kinase-3β (GSK-3β)/PGC-1α pathway, thereby exerting neuroprotective effects in a rotenone-induced PD model (Zhang et al., 2017). Teaghrelin is a peptide analog of growth hormone-releasing hormone extracted from Chin-Shin Oolong tea, which exhibits significant anti-muscle atrophy effects (Hsieh et al., 2020). It can enhance the degeneration of dopaminergic neurons induced by 1-methyl-4-phenyl-1.2,3,6-tetrahydropyridine (MPTP) in mice through the promotion of PINK1/parkin-mediated mitophagy and AMPK/SIRT1/PGC-1α-mediated mitochondrial biogenesis. This indicates its potential as a therapeutic agent for PD (Jhuo et al., 2024).

Luteolin is a naturally occurring flavonoid found in various traditional Chinese herbs, known for its significant anti-inflammatory, antioxidant, and neuroprotective properties. It is considered a candidate drug for the treatment of AD (Jeon et al., 2018). It may enhance mitochondrial biogenesis and Aβ degradation in 3 × Tg-AD mice and primary neurons by improving the expression levels of PGC-1α, Nrf1, Nrf2, and TFAM in the hippocampus of AD mice. Consequently, this action leads to an improvement in cognitive impairments observed in AD mice (He et al., 2023). Myricetin is a natural flavonoid extracted from *Myrica rubra* (Lour.) Siebold and Zucc. It possesses anti-inflammatory and antioxidant activities, and has been demonstrated to protect hippocampal CA3 pyramidal neurons in AD model rats (Ramezani et al., 2016). Furthermore, it can inhibit the phenotypic transformation of microglial cells by promoting the expression of mitochondrial dynamics proteins (FIS1, Drp1, OPA1, Mfn2) and mitochondrial biogenesis proteins (TFAM, Nrf1), thereby improving pathological deposits in 3 × TG-AD mice (Liu et al., 2023).

Arctigenin is a phenylpropanoid dibenzyl butyrolactone lignan extracted from *Arctium lappa* L., known for its pharmacological activities, including anti-inflammatory, antioxidant, and antitumor effects. Its derivatives alleviate gastrocnemius muscle atrophy and motor neuron loss in SOD1 mutant ALS mouse models by promoting mitochondrial biogenesis *via* the AMPK/SIRT1/PGC-1α pathway (Xiong et al., 2024).

**FIGURE 3:**
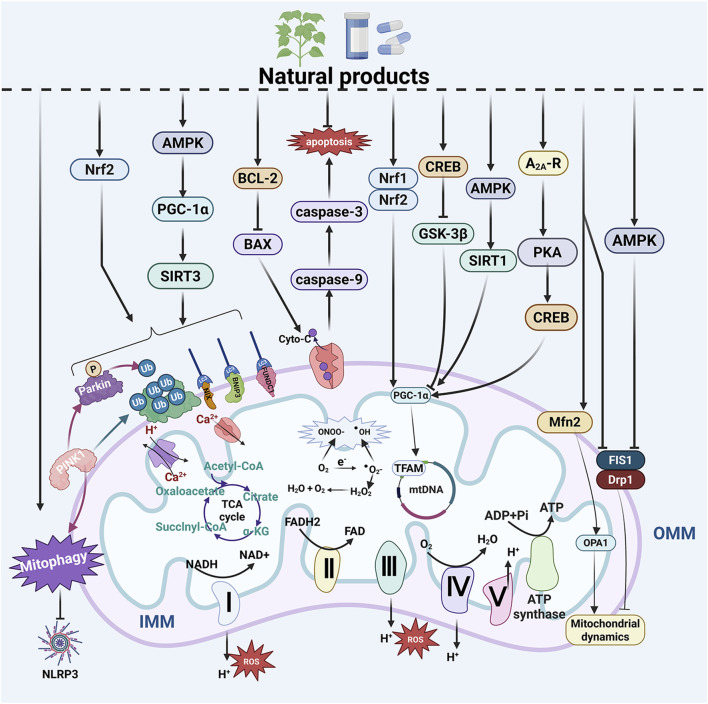
Natural products improve MQC system dysfunction in NDs. The MQC system plays a crucial role in neurodegenerative diseases (NDs). Natural products have the potential to regulate mitochondrial biogenesis, mitophagy, and mitochondrial dynamics. Additionally, they influence mitochondria-dependent apoptosis, maintain mitochondrial calcium homeostasis, modulate mitochondrial genetics, alleviate mitochondrial oxidative stress, and enhance mitochondrial energy metabolism—all of which contribute to the improvement of NDs. ADP, adenosine diphosphate; AMPK, AMP-activated protein kinase; ATP, adenosine triphosphate; A_2A_-R, A2A receptor; BAX, BCL-2 associated X protein; BCL-2, B cell lymphoma 2; BNIP3, BCL2 interacting protein 3; CREB, cAMP response element-binding protein; Cyto-C, cytochrome c; Drp1, dynamin related protein 1; FAD, flavin adenine dinucleotide; FADH, flavine adenine dinucleotide, reduced; FIS1, fission 1 protein; FUNDC1, FUN14 domain-containing protein 1; GSK-3β, glycogen synthase kinase-3β; IMM, inner mitochondrial membrane; LC3, light chain 3; Mfn2, mitofusin 2; mtDNA, mitochondrial DNA; NADH, nicotinamide adenine dinucleotide; NDP52, Nuclear dot protein 52; NIX, NIP3-like protein X; NLRP3, NOD-like receptor thermal protein domain associated protein 3; Nrf1, nuclear respiratory factor 1; Nrf2, nuclear respiratory factor 2; OMM, outer mitochondrial membrane; OPA1, optic atrophy protein 1; PGC-1α, peroxisome proliferator-activated receptor-γ coactivator-1α; PINK1, PTEN-induced putative kinase 1; PKA, protein kinase A; SIRT1, sirtuin 1; SIRT3, sirtuin 3; TFAM, mitochondrial transcription factor A; Ub, ubiquitin; ULK1, Unc-51-like kinase 1.

β-Lapachone is primarily extracted from the roots of the south American Lapacho tree (*Tabebuia avellanedae*) and possesses a variety of pharmacological activities, including anticancer, anti-inflammatory, and wound healing properties. It has been demonstrated to activate mitochondrial biogenesis by promoting the expression of SIRT1, CREB, and PGC-1α in the brains of R6/2 transgenic HD mice. This activation subsequently improves both rota-rod performance and clasping scores in R6/2 transgenic HD mice (Lee M. et al., 2018). *Gastrodia elata* BL. is a commonly referenced Chinese herbal medicine documented in the *Compendium of Materia Medica*. Its active components have been shown to possess neuroprotective effects. Furthermore, its extracts can reduce the aggregation of mHTT in PC12 cells *via* an adenosine A2A receptor (A2A-R)/protein kinase A (PKA)/CREB/PGC-1α-dependent pathway, positioning it as a potential therapeutic target for HD (Huang et al., 2018) (Figure 3; Supplementary Table 1).

### 6.2 Improving mitophagy

Acteoside is a principal active component extracted from the plant *Cistanche deserticola* Ma, belonging to the Scrophulariaceae family. Previous studies have demonstrated its significant inhibitory effects on neuroinflammation and revealed its neuroprotective properties (Gao et al., 2024a). Additionally, research has shown that acteoside can promote PINK1/parkin-mediated mitophagy through the activation of Nrf2, while simultaneously inhibiting neuronal ferroptosis and excessive accumulation of ROS in PD model mice, thereby ameliorating cognitive and behavioral deficits associated with PD models (Han et al., 2024). Hederagenin is a natural triterpenoid saponin primarily found in *Hedera helix* L. It exhibits a range of pharmacological properties, including anti-inflammatory, anticancer, and neuroprotective effects. Notably, it can exert neuroprotective actions against 6-OHDA-induced SH-SY5Y cells by activating mitophagy while concurrently reducing α-Syn expression and excessive accumulation of ROS. These characteristics suggest its potential for the treatment of PD (Li X. et al., 2024). Andrographolide is one of the main active components derived from the Asian medicinal plant *Andrographis paniculata* (Burm. f.) Wall. ex Nees in Wallich. It possesses pharmacological activities such as anti-inflammatory, antitumor, and immunomodulatory effects. Furthermore, it can inhibit microglial activation by activating parkin-mediated mitophagy, thereby mitigating damage to dopaminergic neurons and improving behavioral parameters in a PD model mouse (Ahmed et al., 2021).

Cornuside is a cycloether terpenoid glycoside derived from *Cornus officinalis* Sieb. Et Zucc., which exhibits pharmacological activities including anti-neuroinflammation and antioxidant stress effects. It has been identified as a potential natural product for the treatment of AD (Yu et al., 2023). Futhermore, cornuside alleviates the accumulation of Aβ1-42 in the ventricles of AD mice by activating PINK1/parkin-mediated mitophagy and inhibiting NOD-like receptor thermal protein domain associated protein 3 (NLRP3) inflammasome activation, thereby protecting synapses and neurons from damage (Zhou et al., 2025). Berberine is a bioactive alkaloid extracted from *Coptis chinensis* Franch. It exhibits neuroprotective properties and has been shown to enhance memory and cognitive function. Furthermore, research have indicated that it can reduce oxidative stress, Aβ deposition, and hyperphosphorylation of Tau protein in AD models by promoting mitophagy and alleviating mitochondria-dependent apoptosis (Wang et al., 2023). Aloe-Emodin is an anthraquinone compound predominantly found in the rhizomes of various traditional Chinese herbal medicines, including *Rheum officinale* Baill., *Aloe vera* (L.) Burm. f., and *Senna tora* (L.) Roxb. It exhibits anti-inflammatory and antioxidant properties and has been demonstrated to inhibit the aggregation of pathological proteins associated with AD (Ho et al., 2015). Furthermore, it can enhance mitophagy through the AMPK/PGC-1α/sirtuin 3(SIRT3) pathway, thereby improving hippocampal neuronal damage and cognitive function in APP/presenilin 1(PS1) transgenic mouse models of AD (Wang et al., 2025) (Figure 3; Supplementary Table 1).

### 6.3 Balancing mitochondrial dynamics

Mangiferin is a natural flavonoid glycoside primarily derived from *Mangifera indica* L., exhibiting multifaceted neuroprotective properties in the context of PD (Feng et al., 2019). It has been shown to restore mitochondrial morphology, enhance ATP content within mitochondria, and simultaneously prevent the translocation of Drp1 protein and mitochondrial autophagy damage in the striatal mitochondria of MPTP-induced PD mice. This ultimately alleviates behavioral impairments and dopaminergic neuronal degeneration in these mice (Wang et al., 2022). Andrographolide directly interacts with Drp1, inhibiting its GTPase activity and oligomerization. This interaction prevents excessive mitochondrial fission, ATP depletion, and subsequent cell apoptosis, thereby alleviating motor deficits in a PD model mouse and improving the damage to dopaminergic neurons (Geng et al., 2019).

Ginsenoside is one of the main active components of *Panax ginseng* C. A. Mey., exhibiting significant anti-inflammatory, antioxidant, immune-regulatory, and antitumor properties. Among its active constituents, ginsenoside Rg1 has been demonstrated to possess potential for improving the pathology of AD (Yang et al., 2023). Additionally, it can alleviate the imbalance between mitochondrial fission and fusion by regulating mitochondrial dynamics through the AMPK/Drp1 signaling pathway. This results in a reduction of Aβ accumulation in the hippocampal region of APP/PS1 mice as well as alleviation of synaptic dysfunction (Zhang Y. et al., 2025). Icariin, one of the main active components extracted from the traditional Chinese medicinal herb *Epimedium brevicornu* Maxim., has been shown in previous studies to enhance spatial learning and memory capabilities in AD model rats (Nie et al., 2010). Furthermore, research indicates that Icariin improves mitochondrial transport in 3 × Tg-AD mouse primary hippocampal neurons by inhibiting Drp1 and promoting Mfn2 expression—and this effect may in turn regulate the abnormal expressions of Aβ and phosphorylated Tau (Chen et al., 2016) (Figure 3; Supplementary Table 1).

### 6.4 Inhibition of mitochondria-dependent apoptosis

Aureusidin is found in various Cyperaceae plants and exhibits significant anti-inflammatory and antioxidant activities. It can modulate the expression of pro-apoptotic proteins (BAX, caspase-3, caspase-9, Cyto-C) and anti-apoptotic protein (BCL-2) through mitochondrial pathways. Consequently, it improves the toxicity induced by 6-OHDA in SH-SY5Y cells, demonstrating potential therapeutic effects for PD (Hu et al., 2024). Cordycepin, a natural adenosine analog extracted from *Cordyceps militaris*, exhibits pharmacological properties such as anti-inflammatory, anti-aging, and anti-tumor effects. Previous study has demonstrated that it can effectively protect PC12 cells from 6-OHDA-induced neurotoxicity through antioxidant activity (Olatunji et al., 2016). Additionally, research has shown that while improving mitochondrial damage, cordycepin significantly downregulates BAX and caspase-3 levels and upregulates BCL-2 and BCL-extra large (BCL-XL) expression. Furthermore, it reduces the release of Cyto-C from mitochondria to the cytoplasm, thereby mitigating dopaminergic neuronal damage in PD models (Jiang et al., 2019).

Geniposide, a cyclohexenyl ether glycoside extracted from *Gardenia jasminoides* J. Ellis, exhibits pharmacological activities such as anti-inflammatory, antioxidant, antitumor, and neuroprotective effects. Previous studies have demonstrated that it can enhance cognitive abilities in AD model mice (Lv et al., 2014). Additionally, other research indicates that geniposide may alleviate apoptosis and Aβ-induced neuronal damage by reducing the release of Cyto-C from mitochondria, lowering the BAX/BCL-2 ratio, and inhibiting caspase 3, 9 activity (Zhao et al., 2016). Biochanin A is a phytoestrogen compound primarily found in leguminous plants, such as *Trifolium pratense* and soy. It has previously been utilized in neuroprotective research (Tan et al., 2013). Furthermore, research have demonstrated that Biochanin A can inhibit mitochondrial-mediated release of Cyto-C and the subsequent activation of caspase cascades. This action restores the ratios of BCL-2/BAX and BCL-XL/BAX, thereby mitigating the cytotoxicity and apoptosis induced by Aβ_25-35_ in PC12 cells, suggesting its potential therapeutic effects for AD (Tan and Kim, 2016).

Chrysin is a natural flavonoid extracted from honey and propolis plants, known for its anti-inflammatory and antioxidant properties. Previous studies have demonstrated that it can inhibit neuronal cell death (Izuta et al., 2008). Additionally, research has found that chrysin can mitigate mitochondrial oxidative damage and subsequent apoptosis by upregulating BCL-2 expression while downregulating the expressions of BAX and BAD. This action ultimately leads to an improvement in neurotoxicity within the striatum of 3-NP induced HD rat models (Thangarajan et al., 2016). Naringin is a common flavonoid glycoside found in grapefruit peel, known for its anti-inflammatory, anti-apoptotic, and neuroprotective properties. It can inhibit mitochondrial-dependent apoptosis by upregulating BCL-2 expression and downregulating BAX expression. This mechanism helps to alleviate the neurotoxicity induced by 3-NP in PC12 cells, indicating its potential as a therapeutic agent for HD (Kulasekaran and Ganapasam, 2015) (Figure 3; Supplementary Table 1).

### 6.5 Improving mitochondrial calcium homeostasis

Chrysotoxine is a natural flavonoid compound predominantly found in *Dendrobium chrysotoxum* Lindl. It serves as an effective free radical scavenger and exhibits neuroprotective properties (Song et al., 2012). Furthermore, studies have shown that it can restore mitochondrial calcium homeostasis while reducing BAX/BCL-2-mediated mitochondrial-dependent apoptosis and mtROS accumulation. Consequently, chrysotoxine demonstrates neuroprotective effects on the human neuroblastoma cell line SHSY5Y induced by 6-OHDA, indicating its potential for treating PD (Song et al., 2010).

Urolithin A is a natural metabolite of ellagitannins, a class of compounds found in pomegranates and several other fruits and nuts. It has been shown to cross the BBB and mitigate neurotoxicity induced by Aβ_1-42_ (Yuan et al., 2016). Additionally, research indicates that it can inhibit AhR nuclear translocation-mediated transglutaminase 2 (TGM2) transcription under high-glucose exposure, thereby reducing the interaction between inositol 1,4,5-triphosphate receptor (IP3R1) and VDAC1, mitochondrial calcium influx, mtROS accumulation, Tau phosphorylation, and neuronal cell death. Ultimately, this process alleviates excessive deposition of Aβ and neuronal degeneration under high-glucose conditions (Lee et al., 2021). N,N-dimethyltryptamine is a naturally occurring hallucinogenic alkaloid found in both animals and plants. It can enhance the tricarboxylic acid (TCA) cycle by regulating mitochondrial calcium uptake and facilitating endoplasmic reticulum-mitochondria interactions through the activation of sigma-1 receptor. Additionally, it has been shown to improve cognitive impairment in 3 × Tg-AD mice while reducing Aβ pathological deposition in the hippocampus and prefrontal cortex (Cheng et al., 2024).

Kaempferol is a naturally occurring dietary flavonol found in various vegetables and fruits. It has been shown to activate the mitochondrial Ca^2+^ uniporter, which enhances mitochondrial calcium uptake, alleviates endoplasmic reticulum stress, and restores the function of the mitochondrial electron transport chain. Consequently, this results in improved survival and behavioral characteristics of motor neurons in C9ORF72-ALS model mice (Pilotto et al., 2025) (Figure 3; Supplementary Table 1).

### 6.6 Regulation of mitochondrial genetics

*Centella asiatica* (L.) Urb. is a commonly used traditional Chinese medicinal herb. Its aqueous extract has been shown to alleviate spatial memory deficits and neuronal damage induced by Aβ in mice. Research indicates that its extract can reduce the expression defects of proteins encoded in the mitochondrial ETC and improve mitochondrial dysfunction in the hippocampus. Therefore, it may have a beneficial effect on spatial and contextual memory in AD model mice (Gray et al., 2018). Additionally, research has shown that its water extract can enhance the expression of mitochondrial DNA-derived genes such as Mt-ND1, Mt-CYB, Mt-CO1, and Mt-ATP6. This enhancement restores the normal function of proteins in the electron transport chain (ETC) complexes, improves ATP expression, and alleviates ROS accumulation. Consequently, these effects lead to a reduction in Aβ-induced cytotoxicity in MC65 and SH-SY5Y cells (Gray et al., 2015).

Resveratrol is a naturally occurring polyphenolic compound primarily found in grape skins. It exhibits antioxidant properties and strongly induces mitochondrial activity by increasing the levels of PGC-1α and TFAM. This action ameliorates the expression deficits of mitochondrial ETC-encoded genes, which in turn improves motor impairments and learning functions in HD model mice (Naia et al., 2017) (Figure 3; Supplementary Table 1).

### 6.7 Improving mitochondrial oxidative stress

Auraptene is a 7-geranyloxylated coumarin isolated from citrus fruits, capable of crossing the BBB. It exhibits anticancer and neuroprotective properties, notably inhibiting the loss of dopaminergic neurons (Okuyama et al., 2016). Furthermore, research has indicated that auraptene can alleviate oxidative stress in dopaminergic neurons induced by neurotoxins through the activation of Nrf2 and the induction of antioxidant enzyme gene expression. Additionally, it has been shown to improve motor deficits in MPTP-induced PD mice (Jang et al., 2019). Mogroside V is a natural product extracted from *Siraitia grosvenorii*, known for its antioxidant properties. It has been shown to reduce excessive mitochondrial ROS production and dose-dependently restore mitochondrial membrane potential (MMP) while enhancing ATP production. Consequently, Mogroside V improves motor deficits and dopaminergic neuronal degeneration in PD mice induced by the neurotoxin rotenone (Rot) (Luo et al., 2022).

Myricetin is a natural polyphenolic flavonoid predominantly found in nuts, tea, and red wine. It possesses various properties including antibacterial, anti-inflammatory, anticancer, and antioxidant effects. Notably, it may exhibit effective neuroprotective actions in AD (Semwal et al., 2016). Research has shown that myricetin can inhibit high molecular weight (HMW)-amyloid- oligomer (Aβo)-induced mitochondrial dysfunction by reducing the mitochondrial permeability transition, manganese superoxide dismutase (Mn-SOD), and ATP production. Additionally, it decreases ROS generation within mitochondria while enhancing mitochondrial membrane potential. These mechanisms contribute to mitigating HMW-Aβo-induced neurotoxicity and improving cell viability in SH-SY5Y cells. Therefore, myricetin represents a potential therapeutic agent for AD and other neurodegenerative diseases (Kimura et al., 2021). Honokiol is a biphenolic natural product extracted from *Magnolia officinalis* Rehd. et Wils., which has the ability to cross the BBB. It exhibits pharmacological effects in the treatment of anxiety, epilepsy, and cerebrovascular diseases. Honokiol significantly enhances ATP production and mitigates excessive accumulation of mitochondrial ROS by increasing the expression levels of mitochondrial SIRT3. This action leads to an improvement in Aβo-induced neurotoxicity in hippocampal neurons and alleviates memory deficits in PS1_V97L_ transgenic AD mice (Li et al., 2018).

Lycopene is a potent antioxidant primarily found in tomatoes. Previous study have indicated that it may mediate its protective effects in 3-NP-induced HD through the targeting of nitric oxide pathways (Kumar et al., 2009). Additionally, research has found that it exhibits protective effects against mitochondrial dysfunction and oxidative stress induced by 3-NP. Furthermore, it improves cognitive and behavioral impairments in HD model rats, indicating that lycopene may enhance neuronal degeneration through its antioxidant properties (Sandhir et al., 2010) (Figure 3; Supplementary Table 1).

### 6.8 Improving mitochondrial energy metabolism

Ginsenoside Rd is one of the primary active compounds derived from *P. ginseng* C. A. Mey., exhibiting anti-inflammatory and neuroprotective effects. It has been shown to enhance the expression of mitochondrial respiratory complex I, thereby alleviating ATP depletion induced by methyl-4-phenylpyridinium (MPP^+^). Additionally, ginsenoside Rd mitigates oxidative stress and apoptosis, demonstrating neuroprotective properties in human neuroblastoma SH-SY5Y cells and holding potential for improving PD (Liu et al., 2015).

Rutin is a natural flavonoid that is widely present in various fruits and vegetables. 1t exhibits antioxidant and anti-inflammatory properties in multiple diseases, including diabetes, obesity, and AD. Rutin can enhance mitochondrial OXPHOS, providing sufficient ATP for microglial cells in AD mice to effectively clear Aβ while simultaneously improving learning and memory deficits in these animals (Pan et al., 2019). *Sorghum bicolor* L. is one of the traditional staple foods in China. Its extracts are rich in polyphenols, which can enhance mitochondrial ATP production, increase Δψm, and reduce the generation of mitochondrial ROS, thereby alleviating mitochondrial dysfunction. This action exerts a neuroprotective effect against Aβ aggregation and Tau protein phosphorylation in M17 neuroblastoma cells (Rezaee et al., 2025) (Supplementary Table 1; Figure 3).

## 7 Comparison of natural products

The similarities of the natural products involved in this study are mainly reflected in their core functions and pathological compatibility: At the mechanism level, over 40% of the products activate mitochondrial autophagy through the PINK1/Parkin pathway, approximately 35% clear mtROS through the Nrf2 pathway or antioxidant enzyme activity regulation, and nearly 30% focus on ATP increase and ΔΨm recovery. The core all revolve around the three major mitochondrial regulatory pathways of mitochondrial autophagy, oxidative stress, and energy metabolism. In terms of disease adaptation, PD-related products focus on the balance between autophagy and apoptosis, AD-related products enhance mitochondrial biogenesis and dynamic balance, and ALS/HD products target calcium homeostasis and energy metabolism, all of which are in line with the core pathology of the disease.

The differences are reflected in three aspects: The permeability is significantly differentiated, with 18% being strongly permeable (such as lycopene, berberine), 23% moderately permeable (such as baicalein, resveratrol), and 59% weakly permeable (such as mogroside V), which directly affects the potential of central function. Differentiation of similar structural functions, such as baicalein regulating biogenesis and aureusidin targeting apoptosis in flavonoids. The completeness of verification varies. 62% have specific internal and external data, and 38% have evidence gaps (such as chrysotoxine only *in vitro* experiments).

## 8 Toxicology and adverse effects

No natural product on earth exhibits pharmacological effects without also inducing non-specific off-target effects on normal organs and tissues (Zhang G. et al., 2025). The therapeutic effects of natural products may be accompanied by certain toxicities and side effects. Saikosaponin D, one of the active components extracted from the traditional Chinese herb Radix Bupleuri, has been shown in studies to possess neurotoxic properties. It can inhibit the proliferation of primary neuronal stem/progenitor cells (NSCs/NPCs) derived from the hippocampus and adult neurogenesis by modulating the GSK3β/β-catenin signaling pathways (Qin et al., 2019). Ginsenoside Rg1 is one of the main active components extracted from *P. ginseng* C. A. Mey. Research has shown that it can affect embryonic development in both rats and mice. When embryos are exposed to concentrations of 50 μg/mL and 30 μg/mL, respectively, their overall morphological scores significantly decrease, with rats possibly exhibiting greater sensitivity than mice (Liu et al., 2006). *Ginkgo biloba* L. is a traditional herbal medicine whose potential toxicity has not yet been fully elucidated. Research has demonstrated that five flavonoids derived *G. biloba* L. significantly reduce the viability of human renal tubular epithelial cells and normal human liver cells in a dose-dependent manner, indicating their potential hepatotoxicity and nephrotoxicity (Li et al., 2019). Palmatine is primarily extracted from *Rhizoma Coptidis*, In acute toxicity experiments, the median lethal dose of palmatine was found to be 1,533.68 mg/kg. In subchronic toxicity studies, no deaths or morbidity events associated with palmatine were observed (Yi et al., 2013). Furthermore, research has indicated that palmatine may induce cardiotoxicity in neonatal rats by affecting cardiac myocytes, leading to arrhythmias and cardiac arrest; this toxic response is dose-dependent (Zhang et al., 2018).

Currently, natural products have not gained widespread recognition in the pharmaceutical market and clinical settings. This may be attributed to the fact that the potential toxicity of most natural products has yet to be thoroughly explored. Natural products exhibit pharmacological characteristics involving multiple targets and pathways, allowing them to interact with various targets and pathways within biological systems. However, this interaction can inadvertently affect certain target organs and cells due to their side effects. Therefore, identifying the potential toxicity and adverse reactions of natural products is a crucial first step in the development of natural drugs.

## 9 Discussion and conclusion

This review examines clinical and preclinical studies related to mitochondria and common NDs, revealing that the regulatory roles of mitochondria in these conditions are multidimensional. Key aspects include mitochondrial biogenesis, mitophagy, mitochondrial dynamics, mitochondria-dependent apoptosis, mitochondrial calcium homeostasis, mitochondrial genetics, mitochondrial oxidative stress, and mitochondrial energy metabolism. Notably, there are interactions among these mitochondrial regulatory pathways. Due to their multi-targeted effects, multiple pathways of action, and high safety profiles in pharmacology, natural products are increasingly prevalent in new drug development research. A retrospective analysis of previous studies suggests that enhancing mitochondrial quality control through natural products represents a promising future strategy for the prevention and treatment of NDs.

Despite the broad therapeutic potential of natural products in NDs, several challenges remain: 1. Research on mitochondrial-related NDs is primarily confined to preclinical studies, with limited evidence from de-animalized models; 2. Although various natural products demonstrate significant potential for the prevention and treatment of NDs, their poor stability, low solubility, potential toxicity, and limited permeability across the BBB continue to restrict further clinical applications; 3. There is a lack of techniques such as gene knockout or antagonists to validate the specificity of natural products in regulating various mitochondrial pathways involved in NDs; 4. The potential toxicity of most natural products remains unclear, necessitating further exploration before their formal clinical application.

In summary, natural products exhibit significant potential in the prevention and treatment of NDs through MQC. However, due to the limited number of preclinical studies and insufficient analytical depth, further research is necessary to evaluate the impact of natural products on mitochondrial dysfunction in NDs. Firstly, it is essential to enhance research on delivery systems for natural products to improve their permeability across the BBB, ensuring an effective drug concentration within the central nervous system. For example, the optimization of membrane permeability can be achieved by enhancing the central delivery of weakly permeable products through nanoformulations or structural modifications. Secondly, gene knockout or antagonist-based reverse validation experiments should be employed to explore the underlying mechanisms by which natural products ameliorate mitochondrial dysfunction associated with NDs. Finally, multi-omics technologies need to be utilized to investigate interactions among various mitochondrial regulatory pathways. Moreover, conducting large-scale, multicenter, high-quality double-blind clinical trials is crucial for assessing both the efficacy and safety of natural products in improving neurodegenerative conditions through modulation of mitochondrial pathways.

## Data Availability

The original contributions presented in the study are included in the article/Supplementary Material, further inquiries can be directed to the corresponding authors.

## Glossary

Aβ: myloid-β
Aβo: amyloid-oligomer
AD: Alzheimer’s disease
AMP: adenosine monophosphate
AIF: apoptosis-inducing factor
AMPK: AMP-activated protein kinase
Apaf-1: apoptotic protease activating factor-1
α-Syn: alpha-synuclein
ALS: amyotrophic lateral sclerosis
APP: amyloid precursor protein
ATP: adenosine triphosphate
A2A-R: A2A receptor
BAK: BCL-2 associated K protein
BAX: BCL-2 associated X protein
BBB: blood-brain barrier
BCL-2: B cell lymphoma 2
BCL-XL: BCL-extra large
BDNF: brain-derived neurotrophic factor
BNIP3: BCL2 interacting protein 3
CaMK: calmodulin-dependent protein kinase
CAT: catalase
cGAS: cyclic guanosine monophosphate-adenosine monophosphate synthase
CREB: cAMP response element-binding protein
Cyto-C: cytochrome c
D-loop: displacement loop
Drp1: dynamin related protein 1
Endo G: endonuclease G
ENT: equilibrative nucleoside transporter
ERMCSs: endoplasmic reticulum-mitochondria contact sites
ETC: electron transport chain
FIS1: fission 1 protein
FUNDC1: FUN14 domain-containing protein 1
GSH-PX: glutathione peroxidase/reductase
GSK-3β: glycogen synthase kinase-3β
GTPases: guanosine triphosphate hydrolases
HD: Huntington’s disease
HIV: human immunodeficiency virus
HMW: high molecular weight
HTT: huntingtin gene
IAPs: inhibitors of apoptosis proteins
IFN: type I interferon
IFNAR: IFN-α receptor
IMM: inner mitochondrial membrane
IMS: intermembrane space
IP3R1: inositol 1,4,5-triphosphate receptor
Keap1: Kelch-1ike ECH- associated protein l
LC3: light chain 3
L-OPA1: long-OPA1
MAPK: p38 mitogen-activated protein kinase
MCU: mitochondrial Ca2+ uniporter
Mff: mitochondrial fission factor
Mfn1/2: mitofusin 1 and 2
mHtt: mutant huntingtin protein
Mn-SOD: manganese superoxide dismutase
MPP+: methyl-4-phenylpyridinium
mPTP: mitochondrial permeability transition pore
MPTP: 1-methyl-4-phenyl-1.2,3,6-tetrahydropyridine
MQC: mitochondrial quality control
MS: multiple sclerosis
mtDNA: mitochondrial DNA
mtROS: mitochondrial ROS
NDP52: calcium binding and coiled-coil domain 2
NDs: neurodegenerative diseases
NIX: NIP3-like protein X
NLRP3: NOD-like receptor thermal protein domain associated protein 3
Nrf1: nuclear respiratory factor 1
Nrf2: nuclear respiratory factor 2
NSCs/NPCs: primary neuronal stem/progenitor cells
OMM: outer mitochondrial membrane
OPA1: optic atrophy protein 1
OPTN: optineurin
OXPHOS: oxidative phosphorylation
PD: Parkinson’s disease
PGC-1α: peroxisome proliferator-activated receptor-γ coactivator-1α
PINK1: PTEN-induced putative kinase 1
Pirh2: p53-induced RING-H2 protein
PKA: protein kinase A
PRISMA: Preferred Reporting Items for Systematic Reviews and Meta-Analyses
PRX/Trx: peroxiredoxin/thoredoxin
PS1: presenilin 1
ROS: reactive oxygen species
Rot: rotenone
rRNAs: ribosomal RNAs
SIRT1: sirtuin 1
SIRT3: sirtuin 3
SNpc: substantia nigra pars compacta
SOD1: superoxide dismutase 1
S-OPA1: short-OPA1
TARDBP: TAR DNA-binding protein
Tau: microtubule-associated proteins
TCA: tricarboxylic acid
TFEB: transcription factor EB
TFAM: mitochondrial transcription factor A
TGM2: transglutaminase 2
TH: tyrosine hydroxylase
tRNAs: transfer RNAs
VDACs: voltage-dependent anion channels
ΔΨm: membrane potential
3xTg-AD: triple transgenic Alzheimer’s disease mouse models
3-NP: 3-nitropropionic acid
6-OHDA: 6-hydroxydopamine
