# Supplementary material for: Mitochondrial quality control in neurodegenerative diseases: from molecular mechanisms to natural product therapies
Source: Front Physiol. 2025 Oct 16;16:1695681. doi: 10.3389/fphys.2025.1695681 (PMC12571580; doi:10.3389/fphys.2025.1695681)
Supplement: Supplementary file 1 [file Table1.docx]

**Table 1.** Natural products improve mitochondrial dysfunction in neurodegenerative diseases

| Natural products | Main chemical category | Molecular formula | Main sources | Blood-brain barrier permeability (<-0.3: impermeable; -0.3~0.3: moderate penetration; >0.3: strong permeability) | In vivo/in vitro | Targeted diseases | Modeling method | Main indicators | Biology mechanisms | References |
| --- | --- | --- | --- | --- | --- | --- | --- | --- | --- | --- |
| Baicalein | Flavonoids | C_15_H_10_O_5_ | *Scutellaria baicalensis* Georgi | moderate penetration (-0.05) | In vivo and in vitro | PD | Rotenone-induced PD rat; Rotenone-induced SH-SY5Y cells | CREB↑, GSK-3β↓, PGC-1α↑ | Improving mitochondrial biogenesis by activating the CREB/GSK-3β/PGC-1α pathway | (Zhang et al., 2017) |
| Teaghrelin | Polyphenols | N/A | Chin-Shin Oolong tea | N/A | In vivo | PD | MPTP-induced PD mice | PINK1↑, Parkin↑, AMPK↑, SIRT1↑, PGC-1α↑ | Upregulating mitophagy by activating the PINK1/Parkin pathway and improving mitochondrial biogenesis by activating the AMPK/SIRT1/PGC-1α pathway | (Jhuo et al., 2024) |
| Acteoside | Phenethyl glycosides | C_29_H_36_O_15_ | *Cistanche deserticola* Ma | impermeable (-2.96) | In vivo and in vitro | PD | MPTP-induced PD mice; MPP^+^-induced in SH-SY5Y cells | Nrf2↑, PINK1↑, Parkin↑, mitophagy↑, ferroptosis↓, ROS↓ | Promoting PINK1/ Parkin-mediated mitophagy by activating Nrf2, while inhibiting ferroptosis and excessive accumulation of ROS | (Han et al., 2024) |
| Hederagenin | Pentacyclic triterpenoids | C_30_H_48_O_4_ | *Hedera helix* L. | strong permeability (0.96) | In vivo and in vitro | PD | Transgenic Caenorhabditis elegans (C. elegans) models of PD; 6-OHDA-treated SH-SY5Y cells | PINK1↑, Parkin↑, mitophagy↑, ROS↓ | Exerting neuroprotective effects by activating mitophagy, while reducing the expression of α-Syn and excessive accumulation of ROS | (X. Li et al., 2024) |
| Andrographolide | Diterpene lactones | C_20_H_30_O_5_ | *Andrographis paniculata* (Burm. f.) Wall. ex Nees in Wallich | impermeable (-0.88) | In vivo and in vitro | PD | MPTP-induced PD mice; MPP^+^-induced N9 mouse microglia | Parkin↑, LC3-II/ LC3-I↑, NLRP3↓ | Activating mitophagy by promoting parkin expression while inhibiting microglial activation | (Ahmed et al., 2021) |
| Mangiferin | Flavonoids | C_19_H_18_O_11_ | *Mangifera indica* L. | impermeable (-2.67) | In vitro | PD | MPTP-induced PD mice | PINK1↑, Parkin↑, mitophagy↑, translocation of Drp1↓, ATP↑ | Improving the ATP content in mitochondria while preventing the translocation of Drp1 protein in mitochondria and mitophagy damage | (Wang et al., 2022) |
| Andrographolide | Diterpene lactones | C_20_H_30_O_5_ | *Andrographis paniculata* (Burm. f.) Wall. ex Nees in Wallich | impermeable (-0.88) | In vivo and in vitro | PD | MPTP-induced PD mice; Rotenone-induced SH-SY5Y cells | Translocation of Drp1↓, Interaction of Drp1 between FIS1 and Mfn↓, ATP↑, apoptosis↓ | Inhibiting the GTP enzyme activity and oligomerization of Drp1, preventing excessive mitochondrial fission, ATP depletion and apoptosis | (Geng et al., 2019) |
| Aureusidin | Flavonoids | C_15_H_10_O_6_ | A variety of Cyperaceae plants | impermeable (-0.52) | In vitro | PD | 6-OHDA-induced SH-SY5Y cells | BAX↓, caspase-3↓, caspase-9↓, Cyto-C↓, BCL-2↑ | Regulating the expression of pro-apoptotic proteins and anti-apoptotic proteins through the mitochondrial pathway | (Hu et al., 2024) |
| Cordycepin | Nucleoside antibiotics | C_10_H_13_N_5_O_3_ | *Cordyceps militaris* | impermeable (-1.44) | In vivo | PD | Rotenone-induced PD rat | BAX↓, caspase-3↓, caspase-9↓, Cyto-C↓, BCL-2↑, BCL-XL↑ | Improving mitochondrial damage by regulating mitochondrial apoptosis-related proteins | (Jiang et al., n.d.) |
| Chrysotoxine | Flavonoids | C_18_H_22_O_5_ | *Dendrobium chrysotoxum* Lindl. | N/A | In vitro | PD | 6-OHDA-induced SHSY5Y cells | ROS↓, MAPKs↓, ΔΨm↑, apoptosis↓, Ca^2+^ homeostasis restoration | Restoring mitochondrial Ca^2+^ homeostasis while reducing BAX/BCL-2 mediated mitochondrial-dependent apoptosis and mtROS accumulation | (Song et al., 2010) |
| Auraptene | Coumarin derivatives | C_19_H_22_O_3_ | Citrus fruit | strong permeability (0.64) | In vivo and in vitro | PD | MPTP-induced PD mice; SN4741 cells cultured with rotenone or MPP^+^ | Mitochondrial respiration↑, Nrf2↑, antioxidant enzyme↑, mtROS↓ | Improving mitochondrial function by promoting mitochondrial respiration and eliminating ROS | (Jang et al., 2019) |
| Mogroside V | Glycosides | C_60_H_102_O_29_ | *Siraitia grosvenorii* | impermeable (-6.99) | In vivo and in vitro | PD | Rotenone-induced PD mice; Rotenone-induced SH-SY5Y cells | SIRT3↑, SOD2 acetylation↓, mtROS↓, apoptosis↓ | Improving mitochondrial function by eliminating ROS in mitochondria | (Luo et al., 2022) |
| Ginsenoside Rd | Tetracyclic triterpenoids | C_48_H_82_O_18_ | *Panax ginseng* C. A. Mey. | impermeable (-4.97) | In vivo and in vitro | PD | MPTP-induced PD mice; MPP^+^-induced SH-SY5Y cells | mtROS↓, SOD↑, GPX↑, ATP↑, apoptosis↓ | Improving mitochondrial function by alleviating mitochondrial oxidative stress | (Liu et al., 2015) |
| Luteolin | Flavonoids | C_15_H_10_O_6_ | Widely in a variety of Chinese herbal medicines | impermeable (-0.84) | In vivo and in vitro | AD | Triple transgenic AD mice; Aβ-induced primary neuron | PGC-1α↑, Nrf1↑, Nrf2↑, TFAM↑ | Improving mitochondrial biogenesis by promoting the expression of PGC-1α, Nrf1, Nrf2, and TFAM | (He et al., 2023) |
| Myricetin | Flavonoids | C_15_H_10_O_8_ | *Myrica rubra* (Lour.) Siebold & Zucc. | impermeable (-1.00) | In vivo | AD | Triple transgenic AD mice | TFAM↑, Nrf1↑, FIS1↓, Drp1↓, OPA1↑, Mfn2↑ | Improving mitochondrial biogenesis by increasing TFAM and Nrf1 and improving mitochondrial dynamics by regulating the expressions of FIS1, Drp1, OPA1 and Mfn2 | (Liu et al., 2023) |
| Cornuside | Iridoids | C_24_H_30_O_14_ | *Cornus officinalis* Sieb. Et Zucc. | impermeable (-2.98) | In vivo and in vitro | AD | Aβ_1-42_ -induced PD mice; Aβ_1-42_ -induced BV2 cells | PINK1↑, Parkin↑, mitophagy↑, LC3-II/ LC3-I↑, NLRP3↓, GSDMD↓ | Activating mitophagy by Pink1/Parkin pathway while inhibiting NLRP3 inflammasome and its downstream targets | (Zhou et al., 2025) |
| Berberine | Alkaloids | C_20_H_18_NO_4_+ | *Coptis chinensis* Franch. | strong permeability (0.56) | In vivo and in vitro | AD | D-ribose-induced APP/PS1 mice; D-ribose-induced murine neuroblastoma cell line Neuro2a | PINK1↑, Parkin↑, mitophagy↑, ΔΨm↑, ROS↓, Cytc-C↓, apoptosis↓ | Promoting mitophagy and simultaneously alleviating mitochondrial-dependent apoptosis | (Wang et al., 2023) |
| Aloe-Emodin | Anthraquinones | C_15_H_10_O_5_ | *Rheum officinale* Baill., *Aloe vera* (L.) Burm. f. and *Senna tora* (L.) Roxb., etc | impermeable (-1.07) | In vivo and in vitro | AD | APP/PS1 double transgenic AD mice; Aβ_25-35_-induced HT22 cells | AMPK↑, SIRT3↑, PGC-1α↑, PINK1↑, Parkin↑, mitophagy↑ | Enhancing mitophagy through regulating the AMPK/PGC-1α/SIRT3 pathway | (Wang et al., 2025) |
| Ginsenoside Rg1 | Tetracyclic triterpenoids | C_42_H_72_O_14_ | *Panax ginseng* C. A. Mey. | impermeable (-3.41) | In vivo and in vitro | AD | APP/PS1 double transgenic PD mice; Aβ_1-42_-induced HT22 cells | AMPK↑, Drp1↓, OPA1↓, Mfn1↓, Mfn2↓, ΔΨm↑, ROS↓ | Regulating mitochondrial dynamics through the AMPK/Drp1 signaling pathway to alleviate the imbalance between mitochondrial fission and fusion | (Y. Zhang et al., 2025) |
| Icariin | Flavonoids | C_33_H_40_O_15_ | *Epimedium brevicornu*Maxim. | impermeable (-2.99) | In vitro | AD | Primary hippocampal neurons from 3× transgenic AD mice | Drp1↓, Mfn2↓ | Improving mitochondrial transport by inhibiting Drp1 expression and promoting Mfn2 expression | (Chen et al., 2016) |
| Geniposide | Iridoids | C_17_H_24_O_10_ | *Gardenia jasminoides* J.Ellis | impermeable (-2.61) | In vitro | AD | Aβ_1-42_ induced cortical neuron | BAX/BCL-2↓, caspase-3↓, Cyto-C↓ | Alleviating apoptosis by inhibiting the activity of mitochondrial pro-apoptotic proteins | (Zhao et al., 2016) |
| Biochanin A | Flavonoids | C_16_H_12_O_4_ | Leguminous plants | moderate penetration (0.02) | In vitro | AD | Aβ_1-42_ induced PC12 cell | caspase-3↓, caspase-8↓, caspase-9↓, Cyto-C↓, BCL-2/BAX↑, BCL-XL/BAX↑ | Inhibiting mitochondrial-mediated apoptosis | (Tan and Kim, 2016) |
| Urolithin A | A secondary metabolite of the polyphenol compound ellagitannin | C_13_H_8_O_4_ | Among a variety of fruits and nuts | N/A | In vivo and in vitro | AD | Streptozotocin-induced diabetic mice; High glucose-induced SH-SY5Y cell | TGM2↓, Ca^2+^ homeostasis restoration, mtROS↓ | Improving mitochondrial function by inhibiting mitochondrial calcium influx and mtROS accumulation | (Lee et al., 2021) |
| N, N-dimethyltryptamine | Tryptamines | C_12_H_16_N_2_ | A variety of animals and plants | N/A | In vivo and in vitro | AD | 3xTg-AD mice; Aβ-exposed primary hippocampal neurons | Sig-1r↑, Ca^2+^ homeostasis restoration, ER-mitochondria coupling↑, ER stress↓, ATP↑ | Restoring the ER-mitochondrial crosstalk and mitochondrial calcium homeostasis of neurons by activating Sig-1r | (Cheng et al., 2024) |
| *Centella asiatica* (L.) Urb. extract | N/A | N/A | *Centella asiatica* (L.) Urb. | N/A | In vivo | AD | 5×FAD mice | Mitochondrial antioxidant and synaptic gene expression↑ | Improving hippocampal mitochondrial function by increasing mitochondrialantioxidant and synaptic gene expression | (Gray et al., 2018) |
| Myricetin | Flavonoids | C_15_H_10_O_8_ | *Myrica rubra* (Lour.) Siebold & Zucc. | impermeable (-1.00) | In vitro | AD | HMW-Aβo-induced SH-SY5Y cells | Mn-SOD↑, ATP↑, ΔΨm↑, mtROS↑ | Inhibiting the toxicity of HMW-Aβo by promoting the antioxidant effect of mitochondria | (Kimura et al., 2021) |
| Honokiol | Lignans | C_18_H_18_O_2_ | *Magnolia officinalis* Rehd. et Wils. | strong permeability (0.91) | In vivo and in vitro | AD | PS1_V97L_-transgenic mice; Aβ oligomers-induced hippocampal neurons | SIRT3↑, Mn-SOD↑, ATP↑, mtROS↓, apoptosis↓ | Inhibiting the excessive accumulation of mtROS by promoting the expression of SIRT3 in mitochondria | (Li et al., 2018) |
| Rutin | Flavonoids | C_27_H_30_O_16_ | A variety of vegetables and fruits | impermeable (-2.75) | In vivo and in vitro | AD | APP/PS1 (APPswe/PSEN1dE9) double-transgenic mice or 5×FAD mice; Aβ-induced primary microglia | Mitochondrial OXPHOS↑, ATP↑, neuroinflammation↓ | Promoting mitochondrial-mediated Aβ clearance by improving mitochondrial energy metabolism | (Pan et al., 2019) |
| *Sorghum bicolor* L. extract | N/A | N/A | *Sorghum bicolor* L. | N/A | In vitro | AD | Aβ oligomer- induced human neuroblastoma BE (2)-M17 cells | ΔΨm↑, ATP↑ | Promoting mitochondrial function by improving mitochondrial energy metabolism and restoring ΔΨm | (Rezaee et al., 2025) |
| Kaempferol | Flavonoids | C_15_H_10_O_6_ | A variety of vegetables and fruits | impermeable (-0.55) | In vivo and in vitro | ALS | C9-500 BAC mouse line (FVB/NJ-Tg(C9orf72)500Lpwr/J) carrying a human C9ORF72 gene; iMNs and fibroblast-induced neurons | Ca^2+^ homeostasis restoration, ER-mitochondria coupling↑, ER stress↓, ATP↑ | Regulating ER stress response and mitochondrial calcium homeostasis to save the survival of motor neurons in vitro and in vivo | (Pilotto et al., 2025) |
| Arctigenin | Lignans | C_21_H_24_O_6_ | *Arctium lappa* L. | moderate penetration (0.04) | In vivo | ALS | B6SJL Tg-SOD1*G93A-1Gur/J mice carrying the human SOD1 mutant | AMPK↑, SIRT1↑, PGC-1α↑ | Improving mitochondrial biogenesis by activating the AMPK/SIRT1/PGC-1α pathway | (Xiong et al., 2024) |
| β-Lapachone | Naphthoquinones | C_15_H_16_O_3_ | The roots of the south American Lapacho tree (*Tabebuia avellanedae*) | strong permeability (0.69) | In vivo and in vitro | HD | Transgenic mice of the R6/2 line (B6CBA-Tg(HDexon1)62Gpb/3J, 111 CAGs) | CREB↑, SIRT1↑, PGC-1α↑ | Promoting the expression of SIRT1, CREB and PGC-1α to activate mitochondrial biogenesis | (M. Lee et al., 2018) |
| *Gastrodia elata* BL. extract | N/A | N/A | *Gastrodia elata* BL. | N/A | In vitro | HD | *mHtt* overexpression in PC12 cells | PGC-1α↑, Nrf1↑, Nrf2↑, TFAM↑, CREB↑ | Improving mitochondrial biogenesis by activating the A_2A_-R/ PKA/CREB/PGC-1α pathway | (Huang et al., 2018) |
| Chrysin | Flavonoids | C_15_H_10_O_4_ | Honey and propolis plants | moderate penetration (0.01) | In vivo | HD | 3-NP-induced HD rats | BCL-2↑, BAX↓, BAD↓ | Inhibiting mitochondrial oxidative damage and subsequent apoptosis by regulating the expression of apoptosis-related proteins | (Thangarajan et al., 2016) |
| Naringin | Flavonoids | C_27_H_32_O_14_ | Grapefruit peel | impermeable (-2.99) | In vitro | HD | 3-NP-induced PC12 cell | BCL-2↑, BAX↓, Cyto-C↓ | Inhibiting mitochondrial-dependent apoptosis by up-regulating the expression of BCL-2 and down-regulating the expression of BAX | (Kulasekaran and Ganapasam, 2015) |
| Resveratrol | Polyphenols | C_14_H_12_O_3_ | Grape peel | moderate penetration (-0.01) | In vivo and in vitro | HD | YAC128 mice; HD human lymphoblasts | ETC genes expression↑, mtDNA copy number↑ | Improving the transcription of genes related to mitochondrial function in HD | (Naia et al., 2017) |
| Lycopene | Carotenoids | C_40_H_56_ | Tomatoes | strong permeability (1.44) | In vivo | HD | 3-NP-induced HD rats | Mitochondrial respiration↑, mtROS↓, SOD↑, apoptosis↓ | Improving mitochondrial function by alleviating mitochondrial oxidative stress | (Sandhir et al., 2010) |

Aβ, amyloid-β; Aβo, amyloid-oligomer; AD, Alzheimer's disease; AMPK, AMP-activated protein kinase; APP, amyloid precursor protein; α-Syn, alpha-synuclein; ATP, adenosine triphosphate; BAD, BCL-2-associcated agonist of cell death; BAX, BCL-2 associated X protein; BCL-XL, BCL-extra large; BCL-2, B cell lymphoma 2; CREB, cAMP response element-binding protein; Cyto-C, cytochrome c; Drp1, dynamin related protein 1; ER, endoplasmic reticulum; ETC, electron transport chain; FIS1, fission 1 protein; GPX, glutathione peroxidase; GSDMD, gasdermin D; GSK-3β, glycogen synthase kinase-3β; GTP, guanosine triphosphate; HD, Huntington's disease; Mfn2, mitofusin 2; LC3, light chain 3; MAPK, p38 mitogen-activated protein kinase; Mn-SOD, manganese superoxide dismutase; MPTP, 1-methyl-4-phenyl-1,2,3,6-tetrahydropyridine; MPP^+^, methyl-4-phenylpyridinium; mtDNA, mitochondrial DNA; mtROS, mitochondrial ROS; NLRP3, NOD-like receptor thermal protein domain associated protein 3; Nrf1, nuclear respiratory factor 1; Nrf2, nuclear respiratory factor 2; OPA1, optic atrophy protein 1; OXPHOS, oxidative phosphorylation; PD, Parkinson's disease; PGC-1α, peroxisome proliferator-activated receptor-γ coactivator-1α; PINK1, PTEN-induced putative kinase 1; PKA, protein kinase A; PS1, presenilin 1; ROS, reactive oxygen species; Sig-1r, sigma‑1 receptor; SIRT1, sirtuin 1; SIRT3, sirtuin 3; SOD1, superoxide dismutase 1; TFAM, mitochondrial transcription factor A; TGM2, transglutaminase 2; ΔΨm, membrane potential; 3xTg-AD, triple transgenic Alzheimer's disease mouse models; 3-NP, 3-nitropropionic acid; 5×FAD, transgenic mice with five familial Alzheimer’s disease; 6-OHDA, 6-hydroxydopamine
